# Postglacial migration shaped the genomic diversity and global distribution of the wild ancestor of lager-brewing hybrids

**DOI:** 10.1371/journal.pgen.1008680

**Published:** 2020-04-06

**Authors:** Quinn K. Langdon, David Peris, Juan I. Eizaguirre, Dana A. Opulente, Kelly V. Buh, Kayla Sylvester, Martin Jarzyna, María E. Rodríguez, Christian A. Lopes, Diego Libkind, Chris Todd Hittinger

**Affiliations:** 1 Laboratory of Genetics, J. F. Crow Institute for the Study of Evolution, Wisconsin Energy Institute, Center for Genomic Science Innovation, University of Wisconsin-Madison, Madison, United States of America; 2 DOE Great Lakes Bioenergy Research Center, University of Wisconsin-Madison, Madison, United States of America; 3 Department of Food Biotechnology, Institute of Agrochemistry and Food Technology (IATA), CSIC, Valencia, Spain; 4 Centro de Referencia en Levaduras y Tecnología Cervecera (CRELTEC), Instituto Andino Patagónico de Tecnologías Biológicas y Geoambientales (IPATEC) – CONICET / Universidad Nacional del Comahue, Quintral 1250, Bariloche, Argentina; 5 Instituto de Investigación y Desarrollo en Ingeniería de Procesos, Biotecnología y Energías Alternativas (PROBIEN, CONICET-UNCo), Neuquén, Argentina; The University of North Carolina at Chapel Hill, UNITED STATES

## Abstract

The wild, cold-adapted parent of hybrid lager-brewing yeasts, *Saccharomyces eubayanus*, has a complex and understudied natural history. The exploration of this diversity can be used both to develop new brewing applications and to enlighten our understanding of the dynamics of yeast evolution in the wild. Here, we integrate whole genome sequence and phenotypic data of 200 *S*. *eubayanus* strains, the largest collection known to date. *S*. *eubayanus* has a multilayered population structure, consisting of two major populations that are further structured into six subpopulations. Four of these subpopulations are found exclusively in the Patagonian region of South America; one is found predominantly in Patagonia and sparsely in Oceania and North America; and one is specific to the Holarctic ecozone. Plant host associations differed between subpopulations and between *S*. *eubayanus* and its sister species, *Saccharomyces uvarum*. *S*. *eubayanus* is most abundant and genetically diverse in northern Patagonia, where some locations harbor more genetic diversity than is found outside of South America, suggesting that northern Patagonia east of the Andes was a glacial refugium for this species. All but one subpopulation shows isolation-by-distance, and gene flow between subpopulations is low. However, there are strong signals of ancient and recent outcrossing, including two admixed lineages, one that is sympatric with and one that is mostly isolated from its parental populations. Using our extensive biogeographical data, we build a robust model that predicts all known and a handful of additional regions of the globe that are climatically suitable for *S*. *eubayanus*, including Europe where host accessibility and competitive exclusion by other *Saccharomyces* species may explain its continued elusiveness. We conclude that this industrially relevant species has rich natural diversity with many factors contributing to its complex distribution and natural history.

## Author summary

The mysterious wild parent of hybrid-lager brewing yeasts, *Saccharomyces eubayanus*, has been known for less than 10 years. In this time, it has become clear that lager hybrids arose from a subpopulation that has only been isolated in Tibet and North Carolina, USA; but the global diversity of this species has been less explored. Here, we use whole genome sequencing data for 200 strains (174 newly sequenced) to investigate the genetic diversity and geographical distribution of *S*. *eubayanus*. We find that its extensive wild diversity is largely centered in northern Patagonia, which likely was a glacial refugium for this species as three of six subpopulations are endemic to this region. In contrast, *S*. *eubayanus* is rarely isolated outside of Patagonia. In North America, isolates are dominated by an invasive, near-clonal admixed lineage; the result of an outcrossing and migration event. All subpopulations are well-differentiated, with low gene flow between them. This genetic isolation of subpopulations could be due to ecological factors, such as plant host associations. With modeling, we find that many areas of the world are climatically suitable to *S*. *eubayanus*, including Europe, where it has never been isolated. We propose complex ancestries and rich ecologies underlie the global distribution and diversity of this elusive and industrially important species.

## Introduction

In microbial population genomics, the interplay of human association and natural variation is still poorly understood. The genus *Saccharomyces* is an optimal model to address these questions for eukaryotic microbes, as it contains both partly human-associated species (i.e. *Saccharomyces cerevisiae*) and mostly wild species (e.g. *Saccharomyces paradoxus)*. These two examples also illustrate the complexity of studying yeast population genomics. Much of *S*. *cerevisiae* population structure is admixed, and several lineages show signatures of domestication [1–4]. In contrast, *S*. *paradoxus* is almost exclusively found in the wild and has a population structure that is correlated with geography [5,6]. Pure isolates of their more distant relative *Saccharomyces eubayanus* have only ever been isolated from wild environments; yet, hybridizations between *S*. *cerevisiae* and *S*. *eubayanus* were key innovations that enabled cold fermentation and lager brewing [7–10]. Other hybrids with contributions from *S*. *eubayanus* have been isolated from industrial environments [11–14], indicating that this species has long been playing a role in shaping many fermented products. This association with both natural and domesticated environments makes *S*. *eubayanus* an excellent model where both wild diversity and domestication can be investigated.

Since the discovery of *S*. *eubayanus* in Patagonia [7], this species has received much attention, both for brewing applications and understanding the evolution, ecology, population genomics of the genus *Saccharomyces* [15]. In the years since its discovery, many new globally distributed isolates have been found [16–21]. Prior research has suggested that *S*. *eubayanus* is most abundant and diverse in the Patagonian region of South America, where there are two major populations (Patagonia A/Population A/PA and Patagonia B/Population B/PB) that recent multilocus data suggested are further divided into five subpopulations (PA-1, PA-2, PB-1, PB-2, and PB-3) [21]. There are two early-diverging lineages, West China and Sichuan, which were identified through multilocus data [16] and whose sequence divergences relative to other strains of *S*. *eubayanus* are nearly that of currently recognized species boundaries [20,22,23]. A unique admixed lineage has been found only in North America, which has approximately equal contributions from PA and PB [17,20]. Other isolates from outside Patagonia belong to PB, either the PB-1 subpopulation that is also found in Patagonia [19,20], or a Holarctic-specific subpopulation that includes isolates from Tibet and from North Carolina, USA [16,20]. This Holarctic subpopulation includes the closest known wild relatives of the *S*. *eubayanus* subgenomes of lager-brewing yeasts [16,20].

To explore the geographic distribution, ecological niche, and genomic diversity of this industrially relevant species, here, we present an analysis of whole genome sequencing data for 200 *S*. *eubayanus* strains. This dataset confirms the previously proposed population structure [17,20,21] and extends the analysis to fully explore genomic diversity. Even though *S*. *eubayanus* is genetically diverse and globally distributed, there are not large phenotypic differences between subpopulations. This genomic dataset includes evidence of gene flow and admixture in sympatry, as well as admixture in parapatry or allopatry. While *S*. *eubayanus* has a well-differentiated population structure, isolation by distance occurs within subpopulations that are found globally, as well as within subpopulations restricted to a handful of locations. Much of the genetic diversity is limited to northern Patagonia, but modeling suggests that there are more geographic areas that are climatically suitable for this species, including Europe. *S*. *eubayanus* maintains genetic diversity over several dimensions, including multiple high-diversity sympatric populations and a low-diversity widespread invasive lineage. The diversity and dispersal of this and other [24–26] eukaryotic microbial species mirror observations in plants and animals, including humans, which shows how biogeographical and evolutionary forces can be shared across organismal sizes, big and small.

## Results

### Global and regional *S*. *eubayanus* population structure and ecology

**Fig 1:**
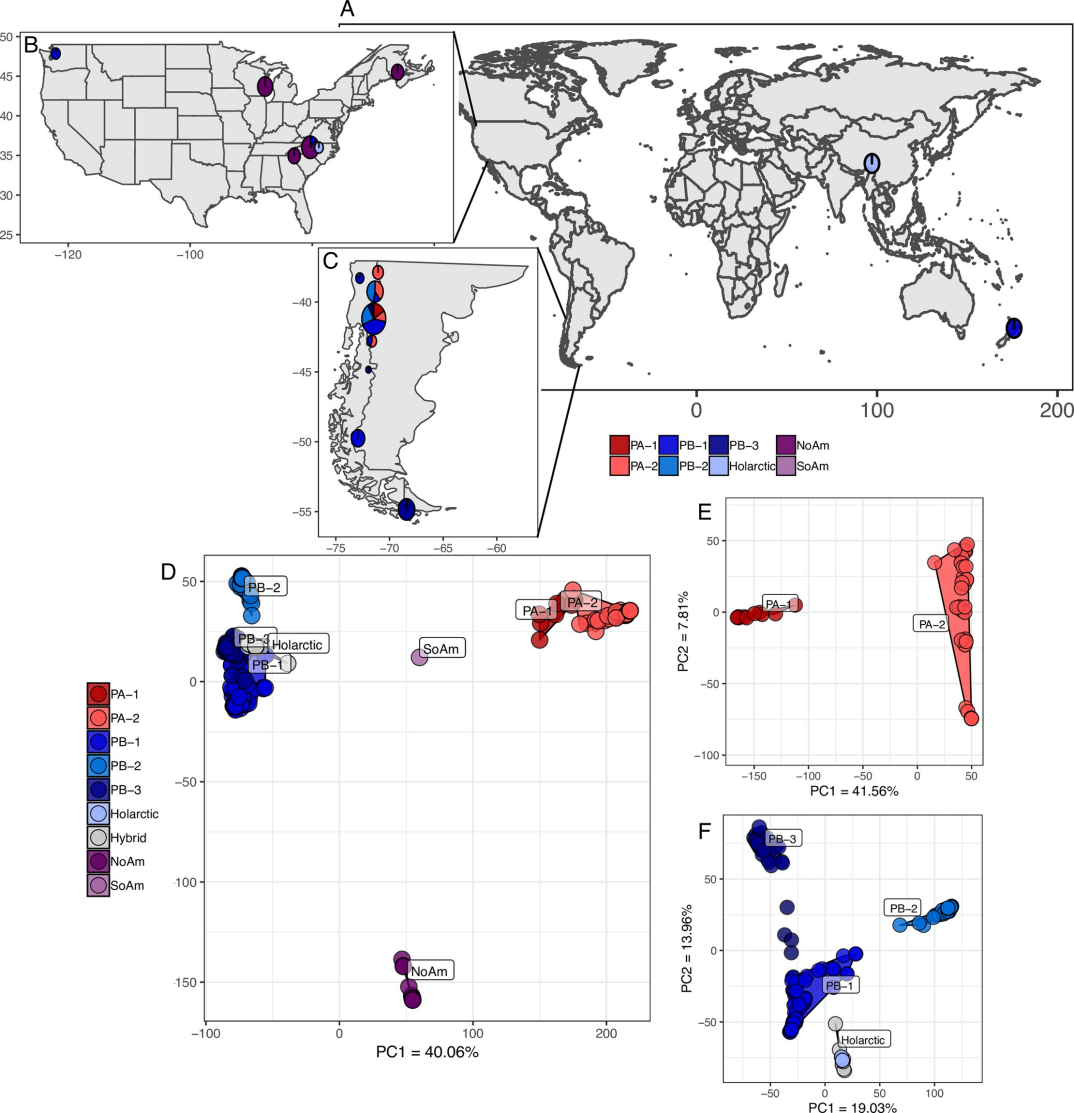
*S*. *eubayanus* distribution and population structure. *S*. *eubayanus* has a global distribution and two major populations with six subpopulations. (A) World map showing isolation locations of *S*. *eubayanus* strains not from North or South America. Points represent one strain colored by subpopulation. Details of sites and subpopulations found in North America (B) and South America (C) with circle size scaled by the number of strains. (D) Whole genome PCA of *S*. *eubayanus* strains and five hybrids with large contributions from *S*. *eubayanus*. (E) PCA of just PA. (F) PCA of just PB and hybrid *S*. *eubayanus* sub-genomes. Color legends in A and D apply to this and all other figures.

To determine population structure, we took several approaches, including Principal Component Analysis (PCA) [28], phylogenomic networks [29], and STRUCTURE-like analyses [30,31]. All methods showed that *S*. *eubayanus* has two large populations that can be further subdivided into a total of six non-admixed subpopulations and one abundant North American admixed lineage (Fig 1D and S1 Fig). We previously described the two major populations, PA and PB-Holarctic [17,20], as well as the subpopulations PA-1, PA-2, PB-1, PB-2, Holarctic, and the North American admixed lineage [20]. PB-3 had been suggested by multilocus data [21], and our new analyses confirm this subpopulation with whole genome sequence data. All of the strains isolated from outside of South America belonged to either the previously described North American admixed lineage (NoAm) or one of two PB subpopulations, PB-1 or Holarctic. This dataset included novel PB-1 isolates from the states of Washington (yHRMV83) and North Carolina (yHKB35). Unexpectedly, from this same site in North Carolina, we also obtained new isolates of the NoAm admixed lineage (Fig 1C and S1 Table), and we obtained additional new NoAm strains in South Carolina. Together, with the North Carolina strains reported here and previously [20], this region near the Blue Ridge Mountains harbors three subpopulations or lineages, PB-1, Holarctic, and NoAm. We were also successful in re-isolating the NoAm lineage from the same Wisconsin site, sampling two years later than what was first reported [17] (S1 Table), indicating that the NoAm admixed lineage is established, not ephemeral, in this location. Additionally, we found one novel South American strain that was admixed between PA (~45%) and PB (~55%) (Fig 1D “SoAm”). This global distribution and the well-differentiated population structure of *S*. *eubayanus* is similar to what has been observed in *S*. *paradoxus* [5,32] and *Saccharomyces uvarum* [11].

*S*. *eubayanus* has been isolated from numerous substrates and hosts, and our large dataset afforded us the power to analyze host and substrate association by subpopulation. We found that PA-2 was associated with the seeds of *Araucaria araucana* (45.71% of isolates, p-val = 6.11E-07, F-statistic = 15.29). Interestingly, while PB-1 was the most frequently isolated subpopulation (34% of isolates), it has never been isolated from *A*. *araucana* seeds. Instead, PB-1 was associated with *Nothofagus antarctica* (52.31% of isolates, p-val = 0.017, F-statistic = 3.10). PB-1 was also the subpopulation isolated the most from *Nothofagus dombeyi* (75% of isolates from this tree species), which is a common host of *S*. *uvarum* [7,21]. PB-2 was positively associated with *Nothofagus pumilio* (36.59% of isolates, p-val = 9.60 E-04, F-statistic = 6.59), which could be an ecological factor keeping PB-2 partly isolated from its sympatric subpopulations, PA-2 and PB-1 (Fig 1C). PB-3 was associated with the fungal parasite *Cyttaria darwinii* (14.29% of isolates, p-val = 0.039, F-statistic = 25.34) and *Nothofagus betuloides* (28.57% of isolates, p-val = 5.02E-06, F-statistic = 60.35), which is only found in southern Patagonia and is vicariant with *N*. *dombeyi*, a host of PB-1. PB-3 was frequently isolated in southern Patagonia (49% of southern isolates) [21], and its association with a southern-distributed tree species could play a role in its geographic range and genetic isolation from the northern subpopulations. Neither *Nothofagus* nor *A*. *araucana* are native to North America, and we found that our North American isolates were from multiple diverse plant hosts, including *Juniperus virginiana*, *Diospyros virginiana*, *Cedrus* sp., and *Pinus* sp. (S1 Table), as well as from both soil and bark samples. In Patagonia, *S*. *eubayanus* has been isolated from exotic *Quercus* trees [21], so even though *Nothofagus* and *A*. *araucana* are common hosts, *S*. *eubayanus* can be found on a variety of hosts and substrates. These observed differences in host and substrate could be playing a role in the maintenance of its population structure, especially in sympatric regions of Patagonia.

### All subpopulations grow at freezing temperatures and on diverse carbon sources

*S*. *eubayanus* comes from a wide range of environments, so we tested if there were phenotypic differences between these subpopulations, focusing on ecologically relevant traits (environmental stress responses) and traits relevant to brewing (growth on different carbon sources). We measured growth rates in liquid media on several carbon sources and recovery from stress responses for a large subset of these strains (190) and 26 lager-brewing strains (S2 and S3 Figs). Lager-brewing strains grew faster on maltotriose than all subpopulations (p-val < 0.05, S2 Fig), which is consistent with this sugar being one of the most abundant in brewing wort but rare in nature [33]. The Holarctic subpopulation grew slower on glucose and maltose compared to all other subpopulations (p-val < 0.05, S2 Fig, S3 Table). Overall, the admixed NoAm lineage performed better than PB-1 (p-val = 0.038, S2 Fig), but there was no interaction with carbon source. Therefore, the admixed lineage’s robustness in many conditions could play a role in its success in far-flung North American sites, including locations where no pure PA or PB strains have ever been found.

Since *S*. *eubayanus*’ contribution to the cold-adaptation of hybrid brewing strains is well established [7,10,34], we measured growth at 0°C, 4°C, 10°C, and 20°C. All subpopulations grew at temperatures as low as 0°C (S2 and S3 Figs), and all *S*. *eubayanus* subpopulations outperformed lager-brewing yeasts (p < 0.05). Within pure *S*. *eubayanus*, there were no temperature-by-subpopulation interactions, indicating that no subpopulation is more cryotolerant than any other subpopulation. In summary, we found that all strains that we tested grew similarly in many environments, and despite the large amount of genotypic diversity observed for this species, we observed much less phenotypic diversity (S2 Fig). Future studies might consider varying other growth conditions, including nitrogen sources, to uncover ecologically relevant differences.

### Subpopulations are well differentiated

The mating strategies and life cycle of *Saccharomyces*, with intratetrad mating and haploselfing, often lead to homozygous diploid individuals [35]. Nonetheless, in *S*. *cerevisiae*, many industrial strains are highly heterozygous [3,4,36]. Here, we analyzed genome-wide heterozygosity in our collection of 200 strains. We found that a vast majority of the strains (85%) had very low heterozygosity (<5% of SNPs called) and nearly all (97%) had less than 10% of variants called as heterozygous (S4B Fig), indicating that most strains in the wild live as homozygous diploids. We found only one individual with more than 20,000 heterozygous SNPs (41% of total SNPs) before filtering for repetitive regions or coverage (S4 Fig). To determine if this high heterozygosity could be due to recent admixture between subpopulations, we phased highly heterozygous regions of its genome and analyzed the two phases separately and found that both phases grouped within PB-1 (S4D Fig). Thus, while this strain is highly heterozygous, it has contributions from only one subpopulation.

This large collection of strains is a powerful resource to explore natural variation and population demography in a wild microbe, so we analyzed several common population genomic statistics in 50-kbp non-overlapping windows across the genome, considering only homozygous SNPs. We found that diversity was similar between subpopulations (S5A Fig). We also calculated Tajima’s D and found that the genome-wide mean was zero or negative for each subpopulation (S5B Fig), which could be indicative of population expansions. In particular, the most numerous and widespread subpopulation, PB-1, had the most negative and consistent Tajima’s D, suggesting a recent population expansion is especially likely in this case.

**Fig 2:**
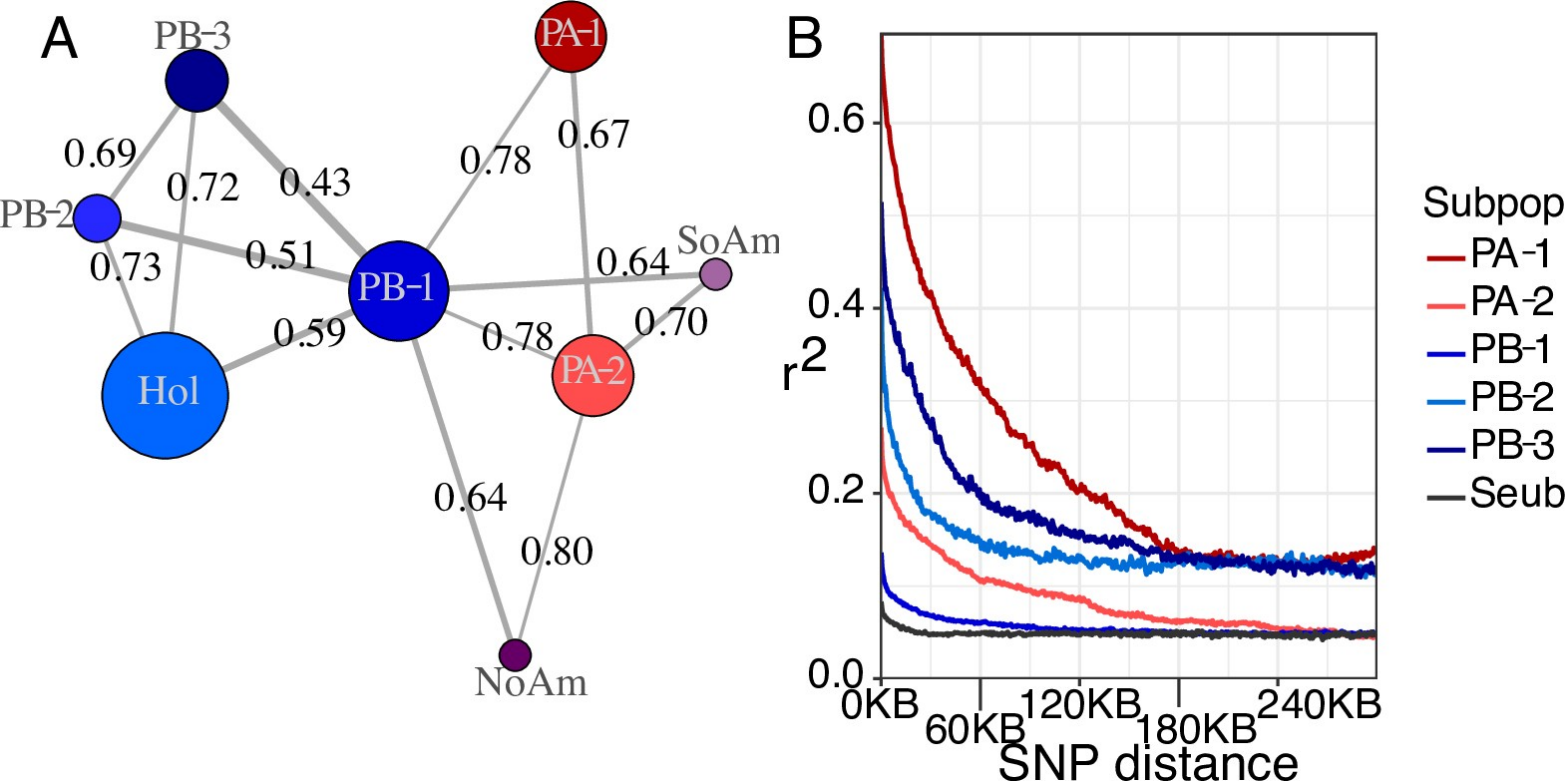
Population genomic parameters. (A) Network built with pairwise F_ST_ values < 0.8 between each subpopulation. F_ST_ values are printed and correspond to line thickness, where lower values are thicker. Circle sizes correspond to genetic diversity. (B) LD decay for each subpopulation (colors) and the species in whole (black).

For the non-admixed lineages, genome-wide average F_ST_ was consistently high across the genome (S5C Fig). In pairwise comparisons of F_ST,_ PB-1 had the lowest values of any subpopulation (Fig 2A, S5D Fig). These pairwise comparisons also showed that, within each population, there has been some gene flow between subpopulations, even though the subpopulations were generally well differentiated. Linkage disequilibrium (LD) decay indicated low recombination in these wild subpopulations (Fig 2B), with variability between subpopulations. The variation between subpopulations could be explained by differing outcrossing rates, population sizes, or a combination thereof. For the species as a whole, LD decayed to one-half at about 5 kbp, which is somewhat higher than the 500bp - 3kbp observed in *S*. *cerevisiae* [1,2,36] and lower than the 9 kbp observed in *S*. *paradoxus* [1], indicating that there is less mating, outcrossing, and/or recombination in this wild species than *S*. *cerevisiae* and more than in *S*. *paradoxus*.

### Recent admixture and historical gene flow between populations

**Fig 3:**
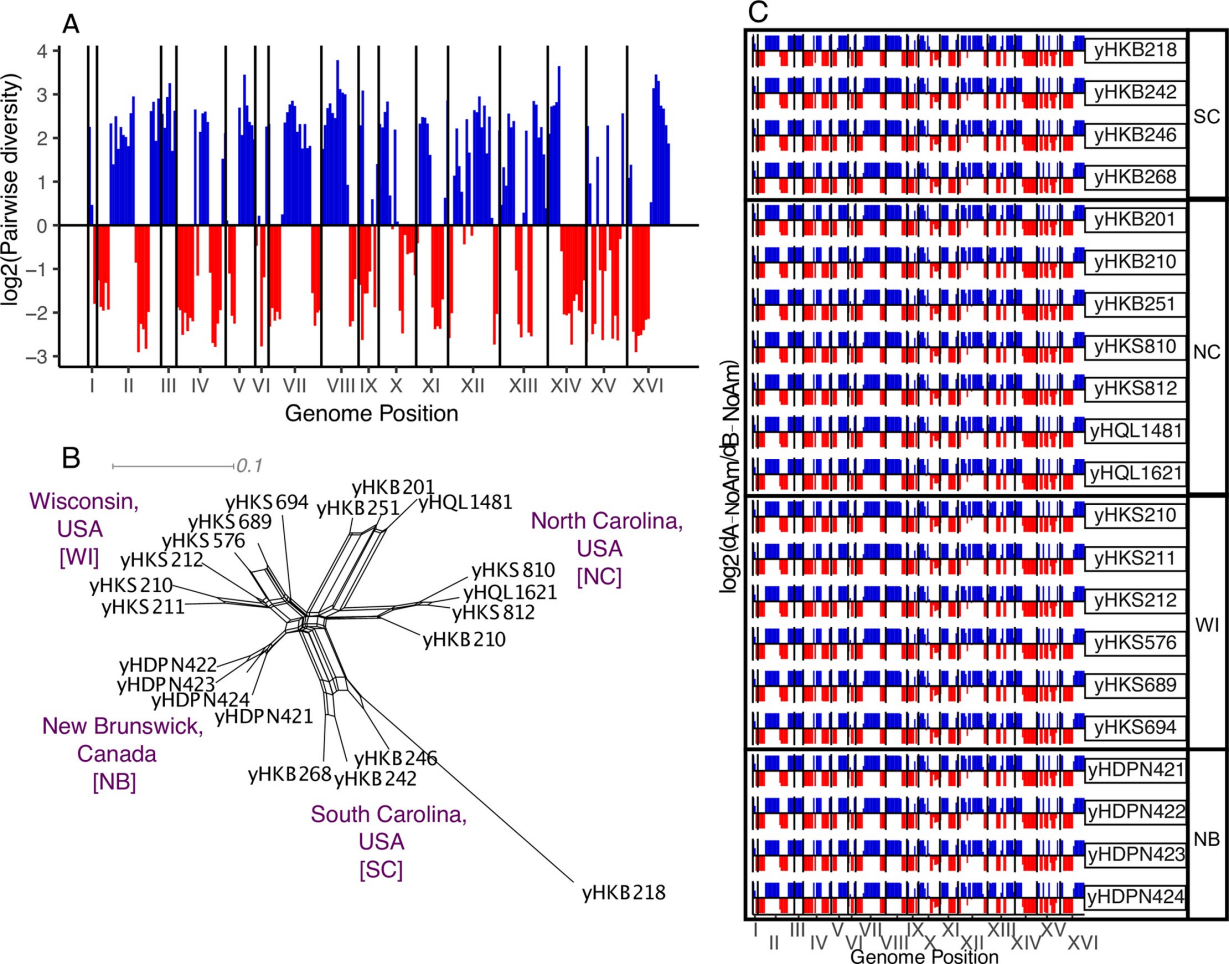
Genomic ancestries of NoAm and SoAm admixed lineages. (A) A representative NoAm strain (yHKS210) log_2_ ratio of the minimum PB-NoAm pairwise nucleotide sequence divergence (dB-NoAm) and the minimum PA-NoAm pairwise nucleotide sequence divergence (dA-NoAm) in 50-kbp windows (adapted from Peris/Langdon et al. 2016) [20]. Colors and log_2_ < 0 or > 0 indicate that part of the genome is more closely related to PA or PB, respectively. (B) Neighbor-Net phylogenetic network reconstructed with the 571 SNPs that differentiate the NoAm strains. The scale bar represents the number of substitutions per site. Collection location is noted in purple. (C) For all 21 NoAm admixed strains, log_2_ ratio of the minimum PB-NoAm pairwise nucleotide sequence divergence (dB-NoAm) and the minimum PA-NoAm pairwise nucleotide sequence divergence (dA-NoAm) in 50-kbp windows. Colors and log_2_ < 0 or > 0 indicate that part of the genome is more closely related to PA or PB, respectively.

We previously reported the existence of 7 strains of an admixed lineage in Wisconsin, USA, and New Brunswick, Canada [17,20]. Here, we present 14 additional isolates of this same admixed lineage. These new isolates were from the same site in Wisconsin, as well as two new locations in North Carolina and South Carolina (S1 Table). Strikingly, all 21 strains shared the exact same genome-wide ancestry profile (Fig 3C), indicating that they all descended from the same outcrossing event between the two main populations of *S*. *eubayanus*. These admixed strains were differentiated by a total of 571 SNPs (<0.005% of the total genome), which also delineated these strains geographically (Fig 3B). Pairwise diversity and F_ST_ comparisons across the genomes suggest that the PA parent came from the PA-2 subpopulation (S7B and S8A Figs) and that the PB parent was from the PB-1 subpopulation (S7C and S8A Figs). Despite the admixed nature of the NoAm strains’ nuclear DNA, we found that this lineage has inherited a mitochondrial genome similar to PA-2 (S6 Fig).

Here, we report a second instance of recent outcrossing between PA and PB. One other strain with fairly equal contributions from the two major populations, PA (~45%) and PB (~55%) (S7D–S7F Fig), was isolated from the eastern side of Nahuel Huapi National Park, an area that is sympatric for all subpopulations found in South America. This strain had a complex ancestry, where both PA-1 and PA-2 contributed to the PA portions of its genome (S7E and S8B Figs), indicating that its PA parent was already admixed between PA-1 and PA-2. As with the NoAm admixed strains, the PB parent was from the PB-1 subpopulation (S7F and S8B Figs). Together, these two admixed lineages show that outcrossing occurs between the two major populations, and that admixture and gene flow are likely ongoing within sympatric regions of South America.

We also found examples of smaller tracts of admixture between PA and PB that were detectable as 2–12% contributions. These introgressed strains included the taxonomic type strain of *S*. *eubayanus* (CBS12357^T^), whose genome sequence was mostly inferred to be from PB-1, but it had a ~4% contribution from PA-1 (S9 Fig). We found several other examples of admixture between PA and PB, as well as admixture between subpopulations of PA or of PB (S4 Table).

In our collection of 200 strains, we observed nuclear genome contributions from *S*. *uvarum* in four strains. These four strains all shared the same introgression of ~150-kbp on chromosome XIV (S10A&S10B Fig). When we analyzed the portion of the genome contributed by *S*. *eubayanus*, we found that these strains were all embedded in the PB-1 subpopulation (S10C Fig). Analysis of the 150-kbp region from *S*. *uvarum* indicated that the closest *S*. *uvarum* population related to these introgressed strains was SA-B (S10D Fig), a population restricted to South America that has not previously been found to contribute to any known interspecies hybrids [11]. These strains thus represent an independent hybridization event between South American lineages of these two sister species that is not related to any known hybridization events among industrial strains [11]. These strains show that *S*. *eubayanus* and *S*. *uvarum* can and do hybridize in the wild, but the limited number (n = 4) of introgressed strains, small introgression size (150-kbp), and shared breakpoints suggest that the persistence of hybrids in the wild is rare. The complexity of the evidence of hybridization between *S*. *eubayanus* and *S*. *uvarum* and within *S*. *eubayanus* between subpopulations make the sister-species *S*. *eubayanus* and *S*. *uvarum* an exciting system to further explore pre- and post-zygotic isolation between microbial organisms.

### Northern Patagonia is a diversity hot spot

**Fig 4:**
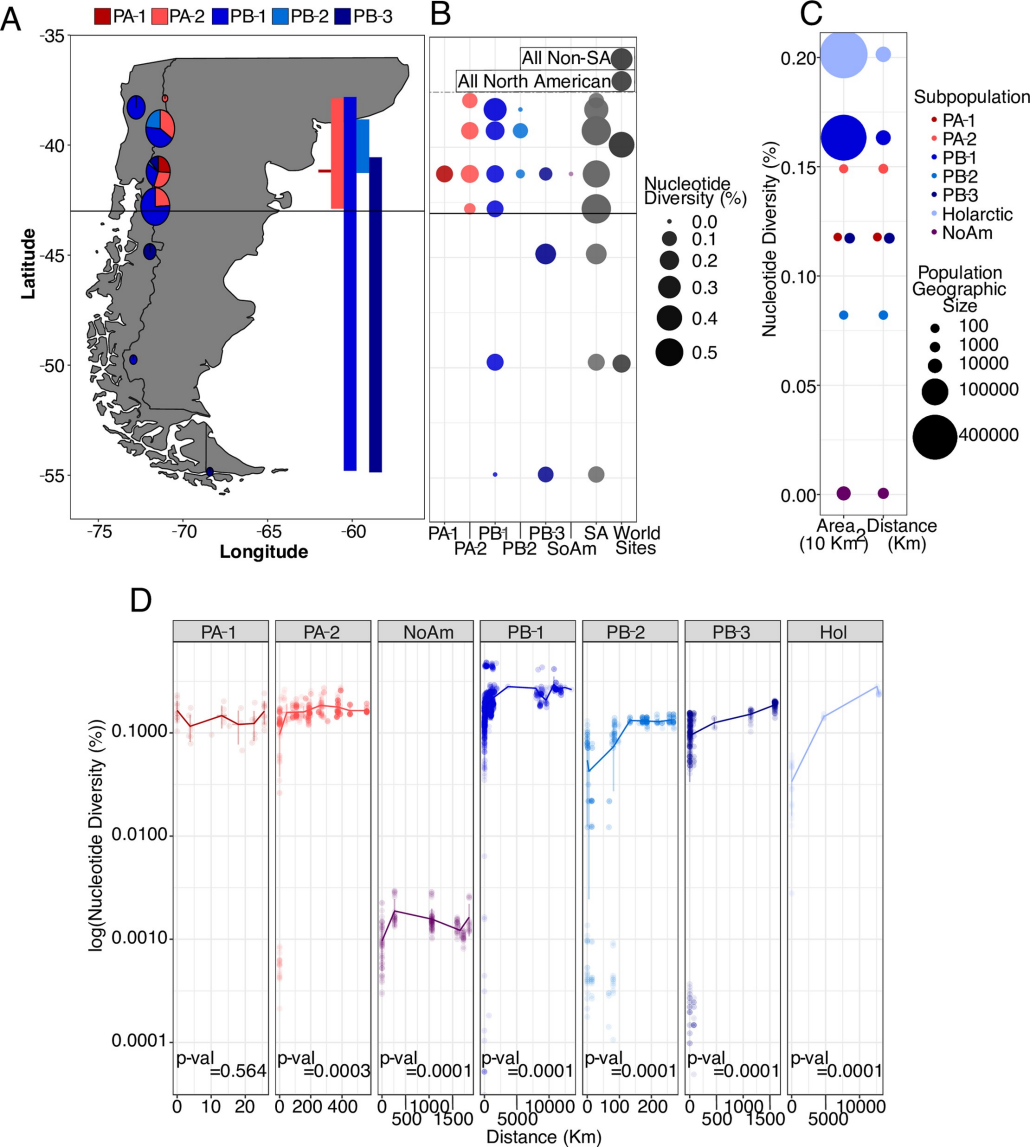
South American genomic diversity versus range, diversity by area, and isolation by distance. (A) Range and genomic diversity of South American sampling sites. Circle sizes correspond to nucleotide diversity of all strains from that site, and pie proportions correspond to each subpopulation’s contribution to 𝜋 at each site. Latitudinal range of each subpopulation is shown to the right. (B) Nucleotide diversity by subpopulation by sampling site, where larger and darker circles indicate more diversity. “SA Sites” in gray show the diversity of all strains found in each South American (SA) site. “World Sites” in darker gray show the nucleotide diversity of all North American or non-South American strains, regardless of subpopulation, compared to South American strains south or north of 43°S, aligned to mean latitude of all strains included in the analysis. (C) Correlation of nucleotide diversity and the area or distance a subpopulation covers. The y-axis shows the nucleotide diversity of each subpopulation, and circle sizes correspond to the geographic sizes of the subpopulations on a log_10_ scale. Note that PA-1 (dark red) is as diverse as PB-3 (dark blue) but encompasses a smaller area. (D) log_10_(pairwise nucleotide diversity) correlated with distance between strains, which demonstrates isolation by distance. Note that y-axes are all scaled the same but not the x-axes. Holarctic includes the *S*. *eubayanus* sub-genome of two lager-brewing strains. S11A Fig shows the individual plots for the NoAm lineage. S11B Fig shows the individual plot of PB-1.

Patagonia harbors the most genetic diversity of *S*. *eubayanus* in our dataset, and four subpopulations were found only there: PA-1, PA-2, PB-2, and PB-3 (Figs 1B and 4A). Therefore, we examined the genetic diversity and range distributions of the isolates from South America more closely. Nahuel Huapi National Park (Argentina) yielded isolates from all five subpopulations found in South America, was the only place where PA-1 was found, and was the location where the SoAm admixed strain was isolated (Fig 4A and 4B). All five sub-populations were found north of 43°S, an important boundary during the last glaciation period that affects many organisms [37–39]. Species-wide, there was more genetic diversity north of this boundary (Fig 4B). In contrast, only PB-1 and PB-3 were found south of 43°S, with both distributions reaching Tierra del Fuego. The southernmost strains were primarily PB-3 (89.7%), but they included two highly admixed PB-1 × PB-3 strains (S1 & S3 Tables).

Despite the limited geographic range of some subpopulations, their genetic diversity was high, and this diversity often did not scale with the geographic area over which they were found (Fig 4C). The widespread distribution of some subpopulations led us to question if there was isolation by distance within a subpopulation (Fig 4D). We used pairwise measures of diversity and geographic distance between each strain and conducted Mantel tests for each subpopulation. All subpopulations showed significant isolation by distance (S5 Table), except PA-1, likely because it had the smallest geographic range (25 km). Even the Mantel test for the least diverse lineage, NoAm, was highly significant (p-val = 0.0001, R^2^ = 0.106), indicating that each location has been evolving independently after their recent shared outcrossing and dispersal event. Through these pairwise analyses, we also detected two strains from Cerro Ñielol, Chile, that were unusually genetically divergent from the rest of PB-1 and could potentially be a novel lineage (S11 Fig).

### Additional global regions are climatically suitable

The sparse but global distribution of *S*. *eubayanus* raises questions about whether other areas of the world could be suitable for this species. Previous studies have also begun to elucidate the distributions of other *Saccharomyces* species using geolocation data or niche modeling [24–26]. Here, we used the maxent environmental niche modeling algorithm implemented in Wallace [40] to model the global climatic suitability for *S*. *eubayanus*, using GPS coordinates of all known *S*. *eubayanus* strains published here and estimates of coordinates for the East Asian isolates [16]. These niche models were built using the WorldClim Bioclims (v1.4), which are based on monthly temperature and rainfall measures, reflecting both annual and seasonal trends, as well as extremes, such as the hottest and coldest quarters. Consideration of how climatic variables affect yeast distributions is being more frequently done [24–26], and building these models allowed us a novel way to explore climatic suitability.

**Fig 5:**
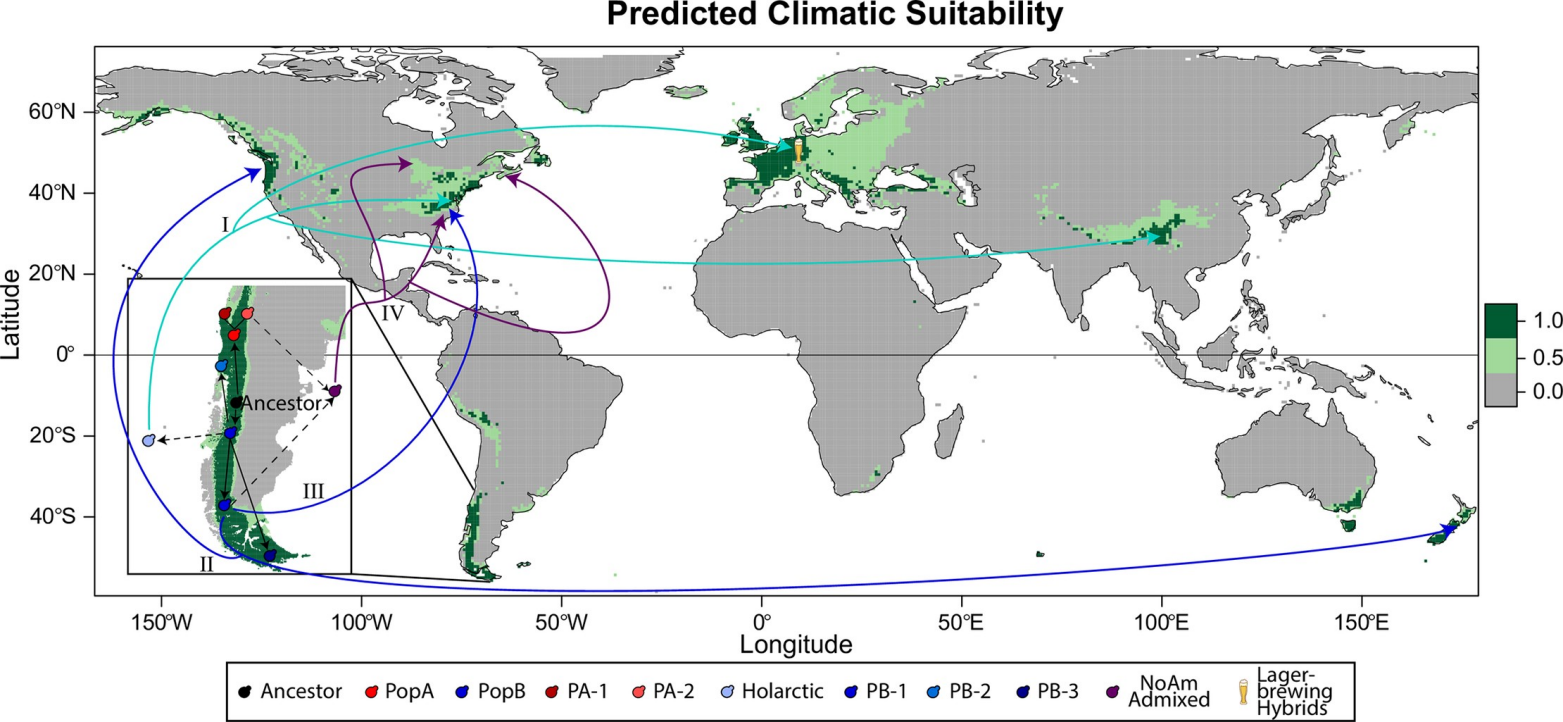
Predicted climatic suitability of *S*. *eubayanus*. Minimum training presence (light green) and 10^th^ percentile training presence (dark green) based on a model that includes all known *S*. *eubayanus* isolations, as well as a scenario of dispersal and diversification out of Patagonia (inset and arrows). Black arrows signify diversification events, dotted lines are diversification events where the population is not found in Patagonia, and colored arrows are migration events for the lineage of matching color. Roman numerals order the potential migration events. *S*. *eubayanus* has not been found in the wild in Europe, but it has contributed to fermentation hybrids, such as lager yeasts. This scenario proposes that the last common ancestor of PA and PB-Holarctic bifurcated into PA (red) and PB-Holarctic (blue), which further radiated into PA-1 (dark red), PA-2 (light red), PB-1 (blue), PB-2 (lighter blue), PB-3 (dark blue), and Holarctic (very light blue). At least four migration events are needed to explain the locations where *S*. *eubayanus* has been found. I. The Holarctic subpopulation was drawn from the PB-Holarctic gene pool and colonized the Holarctic ecozone. II. PB-1 colonized the Pacific Rim, including New Zealand and Washington state, USA. III. An independent dispersal event brought PB-1 to North Carolina, USA. IV. Outcrossing between PA-2 and PB-1 gave rise to a low-diversity admixed linage that has recently invaded a large swath of North America.

Using all known locations of isolation (Fig 5), we found that the best model accurately delineated the known distribution along the Patagonian Andes. In North America, the strains from the Olympic Mountains of Washington state and the Blue Ridge region of North Carolina fell within the predicted areas, and interestingly, these sites had yielded pure PB-1 and Holarctic strains. In contrast, some of the NoAm admixed strains were found in regions that were on the border of suitability in this model (New Brunswick and Wisconsin). In Asia, the model predicts further suitable regions along the Himalayas that are west of known locations.

The uneven global distribution of *S*. *eubayanus* led us to test if models were robust to being built only with the South American locations or only with the non-South American locations (S12 Fig). Remarkably, with just the South American isolates, the model accurately predicted the locations of the non-South American isolates (S12A Fig). Even the model built from the limited number of isolates from outside South America still performed reasonably well, identifying the regions in Patagonia along the Andes where *S*. *eubayanus* has been found (S12B Fig). Collectively, these models and previous work with *S*. *paradoxus* and *S*. *cerevisiae* [26] suggest that climatic modeling can predict other suitable regions for eukaryotic microbes. These approaches could be used to direct future sampling efforts or applied to other microbes to gain further insight into microbial ecology.

Notably, all models agree that Europe is climatically a prime location for *S*. *eubayanus* (S12C Fig), but no pure isolates have ever been found there, only hybrids with *S*. *uvarum*, *S*. *cerevisiae*, or hybrids with even more parents [11,13]. These hybrids with complex ancestries have been found in numerous fermentation environments, suggesting that pure *S*. *eubayanus* once existed, or still exists at low abundance or in obscure locations, in Europe. Thus, the lack of wild isolates from sampling efforts in Europe remains a complex puzzle.

## Discussion

Here, we integrated genomic, geographic, and phenotypic data for 200 strains of *S*. *eubayanus*, the largest collection to date, to gain insight into its world-wide distribution, climatic suitability, and population structure. All the strains belong to the two major populations previously described [17,20], but with the extended dataset, we were able to define considerable additional structure, consisting of six subpopulations and two admixed lineages. These subpopulations have high genetic diversity, high F_ST_, and long LD decay; all measures indicative of large and partly isolated populations undergoing limited gene flow.

Despite the strong population structure, we also observed evidence of admixture. The two recently admixed lineages had nearly equal contributions from the two major populations, but they were the result of independent outcrossing events. The SoAm admixed strain was isolated from a hotspot of diversity and contains contributions from three subpopulations. The NoAm admixed lineage has spread across at least four distant locations, but all strains descended from the same outcrossing event. Since PA has only been isolated in South America, it is intriguing that the NoAm admixed lineage has been successful in so many locations throughout North America. The success of this lineage could be partially explained by its equal or better performance in many environments in comparison to its parental populations (S2 and S3 Figs), perhaps contributing to its invasion of several new locations. Several other Patagonian strains also revealed more modest degrees of gene flow between PA and PB. Finally, we characterized a shared nuclear introgression from *S*. *uvarum* into four Patagonian strains of *S*. *eubayanus*, demonstrating that hybridization and backcrossing between these sister species has occurred in the wild in South America.

*S*. *eubayanus* has a paradoxical biogeographical distribution; it is abundant in Patagonia, but it is sparsely found elsewhere with far-flung isolates from North America, Asia, and Oceania. Most subpopulations displayed isolation by distance, but genetic diversity only scaled with geographic range to a limited extent. In Patagonia, some sampling sites harbor more genetic diversity than all non-Patagonian locations together (Fig 4B). The levels of *S*. *eubayanus* genetic diversity found within northern Patagonia, as well as the restriction of four subpopulations to Patagonia, suggest that Patagonia is the origin of most of the diversity of *S*. *eubayanus*, likely including the last common ancestor of the PA and PB-Holarctic populations.

The simplest scenario to explain the current distribution and diversity of *S*. *eubayanus* is a series of radiations in Patagonia, followed by a handful of out-of-Patagonia migration events (Fig 5). Under this model, PA and PB would have bifurcated in Patagonia, possibly in separate glacial refugia. The east side of the Andes in northern Patagonia was likely the refugium of the PA population, while it is possible that the west side of the Andes in southern Patagonia was the refugium of the PB population [41]. The oldest migration event out of Patagonia would have been the dispersal of the ancestor of the Holarctic subpopulation, drawn from the PB gene pool, to the Northern Hemisphere. Multiple more recent migration events could have resulted in the few PB-1 strains found in New Zealand and the USA. The New Zealand and Washington state strains cluster phylogenetically and could have diversified from the same migration event from Patagonia into the Pacific Rim. The PB-1 strain from North Carolina (yHKB35) is genetically more similar to PB-1 strains from Patagonia, suggesting it arrived in the Northern Hemisphere independently of the Pacific Rim strains. Finally, the NoAm admixed strains are likely the descendants of a single, and relatively recent, out-of-Patagonia dispersal. Given that PA appears to be restricted to northern Patagonia, this region could have been where the hybridization leading to the NoAm lineage occurred. While the dispersal vector that brought this admixed lineage to North America is unknown, its far-flung distribution and low diversity show that it has rapidly succeeded by invading new environments.

Other more complex scenarios could conceivably explain the limited number of strains found outside of Patagonia. For example, PA and PB could represent sequential colonizations of Patagonia from the Northern Hemisphere. Under this model, PA would have arrived first and would then have been restricted to northern Patagonia by competition with the later arrival of PB. The Holarctic subpopulation could be interpreted as remnants of the PB population that did not migrate to Patagonia; but the PB-1 strains from the Northern Hemisphere, especially yHKB35, seem far more likely to have been drawn from a Patagonian gene pool than the other way around. Furthermore, the structuring of the PA-1 and the PA-2 subpopulations and of the PB-1, PB-2, and PB-3 subpopulations are particularly challenging to rectify with models that do not allow for diversification within South America. Even more complex scenarios remain possible, and more sampling and isolation will be required to fully elucidate the distribution of this elusive species and more conclusively reject potential biogeographical models.

*S*. *eubayanus* has a strikingly parallel population structure and genetic diversity to its sister species *S*. *uvarum* [11,20]. Both species are abundant and diverse in Patagonia but can be found globally. Both have early diverging lineages, found in Asia or Australasia, that border on being considered novel species. In South America, both have two major populations, where one of these populations is restricted to northern Patagonia (north of 43°S). However, a major difference between the distribution of these species is that pure strains of *S*. *uvarum* have been found in Europe. Many dimensions of biodiversity could be interacting to bound the distribution and population structure of both *S*. *eubayanus* and *S*. *uvarum*. In particular, we know very little about local ecology, including the biotic community and availability of abiotic resources on a microbial scale, but these factors likely all influence microbial success. We show here that substrate and host association vary between subpopulations. In Patagonia, *S*. *eubayanus* and *S*. *uvarum* are commonly associated with *Nothofagus*, where *N*. *dombeyi* is the preferred host of *S*. *uvarum* [7,21]. Therefore, niche partitioning of host trees could be playing role in the persistence of these species in sympatry in Patagonia. However, in locations where *Nothofagus* is not found and there are perhaps fewer hosts, competitive exclusion could potentially explain why *S*. *eubayanus* has not been found as a pure species in Europe. This competitive exclusion could be caused by *S*. *uvarum*, which shares a similar thermal niche, or by *S*. *paradoxus* or other *Saccharomyces* species that are common in Europe [25,26].

A second factor influencing distribution and population structure could be dispersal. Yeasts could migrate via many avenues, such as wind [42], insect, bird, or other animals [42–45]. Human mediated-dispersal has been inferred for the *S*. *cerevisiae* Wine and Beer lineages and for the *S*. *paradoxus* European/SpA lineage [3–5,46].

A third bounding factor could be a region’s historical climate. Glacial refugia act as reservoirs of isolated genetic diversity that allow expansion into new areas after glacial retreat [47]. In Patagonia, 43°S is a significant geographic boundary due to past geological and climatic variables [21,37], and many other species and genera show a distinction between their northern and southern counterparts, including *Nothofagus* [37,48]. *S*. *eubayanus* and *S*. *uvarum* diversities are also strongly affected by the 43°S boundary [11,21], and it seems likely that the microbes experienced some of the same glaciation effects as their hosts. The strong correlation of *S*. *eubayanus* and *S*. *uvarum* population structures with 43°S further implies a longstanding and intimate association with Patagonia.

The sparse global distribution and complex patterns of genetic diversity continue to raise questions about the niche and potential range of *S*. *eubayanus* and other *Saccharomyces* species [24–26]. Despite extensive sampling efforts, *S*. *eubayanus* has never been isolated in Europe [15]. However, recent environmental sequencing of the fungal specific ITS1 region hinted that *S*. *eubayanus* may exist in the wild in Europe [49]. Considerable caution is warranted in interpreting this result because the rDNA locus quickly fixes to one parent’s allele in interspecies hybrids, there is only a single ITS1 SNP between *S*. *uvarum* and *S*. *eubayanus*, and the dataset contained very few reads that mapped to *S*. *eubayanus*. Still, the prevalence of hybrids with contributions from the Holarctic lineage of *S*. *eubayanus* found in Europe [20] suggests that the Holarctic lineage exists in Europe, or at least existed historically, allowing it to contribute to many independent hybridization events.

The patterns of radiation and dispersal observed here mirror the dynamics of evolution found in other organisms [50,51], including humans [52]. *S*. *eubayanus* and humans harbor diverse and structured populations in Patagonia and sub-Saharan Africa, respectively. In these endemic regions, both species show signals of ancient and recent admixture between these structured populations. Both species have successfully colonized wide swaths of the globe, with the consequence of repeated bottlenecks in genetic diversity. While anatomically modern humans underwent a single major out-of-Africa migration that led to the peopling of the world [52], *S*. *eubayanus* has experienced several migration events from different populations that have led to more punctate global distribution. For both species, intraspecific admixture and interspecific hybridization appear to have played adaptive roles in the success of colonizing these new locations. In humans, introgressions from past hybridizations with both Neanderthals and Denisovans underlie adaptive traits [53], while the cold fermentation of lager-brewing would not be possible without the cryotolerance of *S*. *eubayanus* and the aggressive fermentation of domesticated ale strains of *S*. *cerevisiae* [8]. These parallels illustrate how the biogeographical and evolutionary dynamics observed in plants and animals also shape microbial diversity. As yeast ecology and population genomics [54,55] move beyond the Baas-Becking “Everything is everywhere” hypothesis of microbial ecology [56,57], the rich dynamics of natural diversity that is hidden in the soil at our feet is being uncovered.

## Methods

### Wild strain isolations

All South American isolates were sampled, isolated, and identified as described previously [7,21]. These sampling efforts were focused on the Argentinian region of Patagonia east of the Andes, but also included two sampling sites in Chile. This Patagonian sampling scheme had a *S*. *eubayanus* isolation frequency of ~30%, as previously reported in Eizaguirre et al. (2018) [21]. North American isolates new to this publication were from soil or bark samples from the American states of Washington, Wisconsin, North Carolina, and South Carolina (S1 Table). Strain enrichment and isolation was done as previously described [17,20,58], with a few exceptions in temperature and carbon source of isolation (S1 Table). Specifically, two strains were isolated at 4°C, eight strains were isolated at room temperature, and six strains were isolated on a non-glucose carbon source: three in galactose, two in sucrose, and one in maltose (S1 Table). The isolates from the United States of America were part of the Hittinger Lab’s Wild Yeast Exploration and Analysis Science Team (YEAST) Program, an undergraduate-driven project, which seeks to isolate a wide range of yeasts from diverse substrates [58]. This project has collected over 1,000 samples, but only ~1% of these have yielded *S*. *eubayanus* strains, a rate of North American isolation similar to what was reported in Eizaguirre et al. (2018) [21]. We have attempted to limit our isolation bias by using a wide range of temperature and carbon sources for our isolations. While the NoAm admixed lineage is the most frequently isolated subpopulation in North America, one site in North Carolina yielded both NoAm strains and a pure PB-1 strain.

### Ecological analysis

To determine if there was any association with isolation substrate, we limited our analyses to populations with three or more isolates, by removing the Holarctic subpopulation, and removed any strains with unknown collection information. We were able to categorize the strains as coming from one of five substrates: soil, bark, leaves, seed, or mushroom. For most strains, we also had plant association information (e.g. the plant species providing the bark, leaves, or seed or the plant species nearest to the soil or mushroom). Some strains were isolated from *Cyttaria* sp. mushrooms on trees, so for these we had information about two hosts: the tree species and the fungal species. For analyses of host tree, we did not differentiate by seed, bark, leaves, or soil. We limited our analyses to only include host species that had at least five isolates. We performed a Fisher’s Exact Test with a Bonferroni correction at both the tree host level and at the mushroom host level. All statistical tests were done with R. The test outputs can be found in S2 Table.

### Whole genome sequencing and SNP-calling

Whole genome sequencing was completed with Illumina paired-end reads as described previously [20,59]. Reference mapping, variant calling, and processing files for downstream analyses were done as described previously [20]. Briefly, reads were aligned to the reference genome [60], and SNPs were called. We then masked for repetitive regions, coverage (both low and high), and heterozygosity. Most strains had low heterozygosity (<5% of SNPs called); therefore, very little information was lost by only looking at homozygous variants. Only one strain, yHCT75, had more than 20,000 heterozygous SNPs called (before masking for coverage and repetitive regions). To determine if this high heterozygosity could be due to recent admixture, we pseudo-phased this strain’s data using read-backed phasing in GATK [61] and split SNPs into two phases to check the population of each phase. Short-read data is deposited in the NCBI Short Read Archive under PRJNA555221.

### Population genomic analyses

Population structure was defined using several approaches: fastSTRUCTURE [31], fineSTRUCTURE [30], SplitsTree v4 [29], and Principal Component Analysis with the *adegenet* package in R [28]. fineSTRUCTURE analysis was completed using all strains and 11994 SNPs. The SplitsTree network was built with this same set of strains and SNPs. fastStructure analysis was completed with as subsample of 5 NoAm strains and 150165 SNPs. We tested K = 1 through K = 10 and selected K = 6 using the “chooseK.py” command in fastSTRUCTURE. All calculations of pairwise divergence, F_ST_, and Tajima’s D for subpopulations were computed using the R package *PopGenome* [62] in non-overlapping windows of 50-kbp. Pairwise divergence between strains was calculated across the whole genome using *PopGenome*. LD was calculated using PopLDdecay [63]. Geographic area and distance of subpopulations was calculated using the *geosphere* package in R [64]. The Mantel tests were completed using *ade4* package of R [65]. The F_ST_ network was built with *iGraph* in R [66].

### Niche projection with Wallace

Climatic modeling of *S*. *eubayanus* was completed using the R package *Wallace* [40]. Three sets of occurrence data were tested: one that included only GPS coordinates for strains from South America, one that included only non-South American isolates, and one that included all known isolates (S1 Table). We could use exact GPS coordinates for most strains, except for the strains from East Asia, where we estimated the locations [16]. WorldClim (v1.4) bioclimatic variables were obtained at a resolution of 2.5 arcmin. The background extent was set to “Minimum convex polygon” with a 0.5-degree buffer distance and 10,000 background points were sampled. We used block spatial partitioning. The model was built using the Maxent algorithm, using the feature classes: L (linear), LQ (linear quadradic), H (Hinge), LQH, and LQHP (Linear Quadradic Hinge Product) with 1–3 regularization multipliers and the multiplier step value set to 1. The model was chosen based on the Akaike Information Criterion (AIC) score (S5 Table). With this method, all bioclimatic variables are included. In the final model, different variables were more predictive for different regions, but there was no single variable that was most predictive for all regions. The best models were then projected to the all continents, except Antarctica.

### Phenotyping

Strains were first revived in liquid Yeast Peptone Dextrose (YPD) and grown for 3 days at room temperature. These saturated cultures were then transferred to two 96-well microtiter plates, for growth rate and stress tolerance phenotyping. These plates were incubated overnight. Cells were pinned from these plates into plates for growth rate measurements. For temperature growth assays, cells were pinned into four fresh liquid YPD microtiter plates and then incubated at 0°C, 4°C, 10°C, and 20°C. For the microtiter plates at 0°C, 4°C, and 10°C, OD was measured at least once a day for two weeks or until a majority of the strains had reached stationary phase. Growth on different carbon sources was measured at 20°C in liquid MM media with 2% of the respective carbon source. Carbon sources tested were: glucose, galactose, raffinose, maltose, maltotriose, ethanol, and glycerol. OD was read every two hours for one week or until saturation. All phenotyping was completed in biological triplicate. The carbon source data was truncated to 125 hours to remove artifacts due to evaporation. Growth curves were analyzed using the package *grofit* [67] in R to measure saturation and growth rate. We then averaged each strain over the triplicates. We used an ANOVA corrected with Tukey’s HSD to test for growth rate interactions between subpopulation and carbon source or subpopulation and temperature. We used the R package *pvclust* [68] to cluster and build heatmaps of growth rate by subpopulation.

To test for environmental stress tolerance, we tested recovery from heat shock and from a freeze-thaw cycle. Heat shock was completed by pelleting 200μl saturated culture, removing supernatant, resuspending in 200μl liquid YPD pre-heated to 37°C, and incubating for one hour at 37°C, with a room temperature control. Freeze-thaw tolerance was tested by placing saturated liquid YPD cultures in a dry ice ethanol bath for two hours, with a control that was incubated on ice. After stress, the strains were serially diluted 1:10 and pinned onto solid YPD. These dilution plates were then photographed after 6 and 18 hours. CellProfiler [69] was used to calculate the colony sizes after 18 hours, and the 3^rd^ (1:1000) dilutions were used for downstream analyses. Colony size was averaged over triplicates and normalized by room temperature controls for heat shock and by ice incubation controls for freeze-thaw tolerance. Statistical interactions of subpopulations and stress responses were tested as above. No interactions were significant, so these tests were not reported in the Results section, but are provided in S3 Table and S3A Fig.

## Supporting information

S1 Fig Additional visualizations of population structure. (A) SplitsTree network tree built with 11994 SNPs with subpopulations circled and labeled. (B) FineStructure co-ancestry plot built with 11994 SNPs. Bluer colors correspond to more genetic similarity. Boxes have been added to label the subpopulations. (C) FastSTRUCTURE plot (K = 6) built with 150165 SNPs and showing the same six monophyletic subpopulations found with other approaches. Only five NoAm strains were included in the fastSTRUCTURE analysis.
(TIF)

S2 Fig Phenotypic differences. (A) Heat map of mean of maximum growth rate (change in OD/hour) (GR) on different carbon sources by subpopulation. Warmer colors designate faster growth. (B) Heat map of log_10_ normalized growth at different temperatures by subpopulation.
(TIF)

S3 Fig Additional phenotypic data. (A) Violin plots of recovery from stress, normalized by controls. There were no significant subpopulation-by-stress interactions. (B) Violin plots of log_10_ normalized mean growth rates of each subpopulation at 0°C, 4°C, 10°C, and 20°C. * = p-val < 0.05 of interactions between Lager and PA-2, PB-2, and PB-3 at 10°C; Lager and PA-1, PA-2, PB-1, PB-2, and PB-3 at 20°C; and PB-2 and both PA-2 and NoAm at 20°C. (C) Violin plots of mean growth rate on different carbon sources (* = p-val < 0.05). (D) Heatmaps of significant subpopulation-by-temperature interactions and (E) significant subpopulation-by-carbon-source interactions. Warmer colors indicate that the subpopulation-by-temperature or the subpopulation-by-carbon source interactions on the left hand had a faster growth rate than the subpopulation-by-temperature or the subpopulation-by-carbon source along the bottom; cooler colors represent the reverse. Non-significant interactions, based on multiple test corrections, are in white. More intense colors represent smaller p-values.
(TIF)

S4 Fig Heterozygosity analyses. (A) Summary of all SNPs versus SNPs called as heterozygous included in analyses compared to the taxonomic type strain for all pure *S*. *eubayanus* strains included in this study. Variants were called on a genome that was not repeat-masked, and strains were subsequently masked for high- and low-coverage regions and for repeats. Shown here are SNP counts after masking for coverage and repeat regions. The upper limit of the bar is the total SNP count. The lower point corresponds to SNPs called as heterozygous. The horizontal line is 20k SNPs. The three strains with low SNP calls (on the left) are derived from the type strain. (B) Percent of SNPs called as heterozygous for all wild pure *S*. *eubayanus* strains. The horizontal line is 5% of SNPs called as heterozygous. Most strains have low heterozygosity. (C) Strain yHCT75 (CRUB 1946) is the only strain with > 20K heterozygous SNPs (pre-masking). (D) When the heterozygous SNPs of yHCT75 were pseudo-phased (labeled), both phases clustered with PB-1.
(TIF)

S5 Fig Additional population genomic statistics. (A) Mean pairwise nucleotide diversity (𝜋 * 100) for each subpopulation across the genome in 50-kbp windows. (B) Tajima’s D across the genome in 50-kbp windows for each subpopulation. (C) Mean F_ST_ in 50-kpb windows for each subpopulation compared to all subpopulations. (D) Pairwise F_ST_ for each subpopulation compared to PB-1.
(TIF)

S6 Fig Mitochondrial genome phylogenetic analysis. SplitsTree network tree built with 2199 SNPs from the mitochondrial genome. Subpopulations are labeled. Strain yHCT98 was removed due to poor mitochondrial mapping.
(TIF)

S7 Fig (A) A representative NoAm strain (yHKS210) log_2_ ratio of the minimum PB-NoAm pairwise nucleotide sequence divergence (dB-NoAm) and the minimum PA-NoAm pairwise nucleotide sequence divergence (dA-NoAm) in 50-kbp windows (adapted from Peris/Langdon et al. 2016) [20]. Colors and log_2_ < 0 or > 0 indicate that part of the genome is more closely related to PA or PB, respectively. (B) Pairwise nucleotide sequence divergence of the NoAm strain yHKS210 compared to strains from the PA-1 and PA-2 subpopulations of PA in 50-kbp windows. (C) Pairwise nucleotide sequence divergence of the NoAm strain yHKS210 compared to strains from the PB-1, PB-2, and PB-3 subpopulations of PB in 50-kbp windows. (D) log_2_ ratio of the minimum PB-SoAm pairwise nucleotide sequence divergence (dB-SoAm) and the minimum PA-SoAm pairwise nucleotide sequence divergence (dA-SoAm) in 50-kbp windows. Colors are as in A. (E) Pairwise nucleotide sequence divergence of the SoAm strain compared to strains from the PA-1 and PA-2 subpopulations of PA in 50-kbp windows. (F) Pairwise nucleotide sequence divergence of the SoAm strain compared to strains of the PB-1, PB-2, and PB-3 subpopulations of PB in 50-kbp windows.
(TIF)

S8 Fig Pairwise F_ST_ plots for NoAm and SoAm compared to all other subpopulations. Pairwise F_ST_ for the NoAm lineage (A) or SoAm strain (B) compared to all other subpopulations.
(TIF)

S9 Fig The taxonomic type strain has a mosaic genome. (A) Pairwise genetic divergence of the taxonomic type strain compared to each subpopulation. (B) Comparison of pairwise genetic divergence of the taxonomic type strain compared to PA-1 and PB-1. (C) log_2_ divergence plot (as in Fig 4) showing regions introgressed from PA-1 in the taxonomic type strain.
(TIF)

S10 Fig Four *S*. *eubayanus* strains with *S*. *uvarum* nuclear introgressions. (A) Depth of coverage plots of reads from four strains mapped to both the *S*. *uvarum* (Suva) and *S*. *eubayanus* (Seub) reference genomes. (B) Zoom-in of region on Chromosome XIV where these four strains have the same *S*. *uvarum* (purple) introgression into a *S*. *eubayanus* background. (C) A PCA plot shows that these four strains belong to the PB-1 subpopulation of *S*. *eubayanus*. (D) A PCA plot shows that the introgressed region from *S*. *uvarum* came from the South American SA-B subpopulation of *S*. *uvarum*.
(TIF)

S11 Fig Isolation by distance plots for NoAm and PB-1. (A) Isolation by distance for all NoAm strains. The y-axis has been rescaled compared to Fig 5 for better visualization. (B) Isolation by distance for subpopulation PB-1. Comparisons with strains from Cerro Ñielol are labeled. All comparisons of South American strains with non-South American strains are on the right side.
(TIF)

S12 Fig Additional Wallace climatic models. (A) Model built using only South American isolation locations. (B) Model built using only non-South American sites. (C) Comparison of models based on all known *S*. *eubayanus* collection sites, only South American, or only non-South American sites. Where the models agree is in dark green, where two models agree is in medium green, and where one model predicts suitability is in light green.
(TIF)

S1 Table Collection information for all strains whose genomes were sequenced or analyzed in this study. Where two hosts are given, the strain was isolated a fungal *Cyttaria* species growing on a *Nothofagus* tree.
(XLSX)

S2 Table Ecological analysis output. Bonferroni-corrected Fisher’s Exact Test results of host and subpopulation association.
(XLSX)

S3 Table Average triplicate growth rates for various temperatures and carbon sources. Note that this spreadsheet has multiple sheets.
(XLSX)

S4 Table K = 6 output of FastSTRUCTURE. (XLSX)

S5 Table Mantel test results. (XLSX)

S6 Table Input and output for Wallace climatic modeling. (XLSX)
