# Supplementary figures and images for: Postglacial migration shaped the genomic diversity and global distribution of the wild ancestor of lager-brewing hybrids
Source: PLoS Genet. 2020 Apr 6;16(4):e1008680. doi: 10.1371/journal.pgen.1008680 (PMC7162524; doi:10.1371/journal.pgen.1008680)

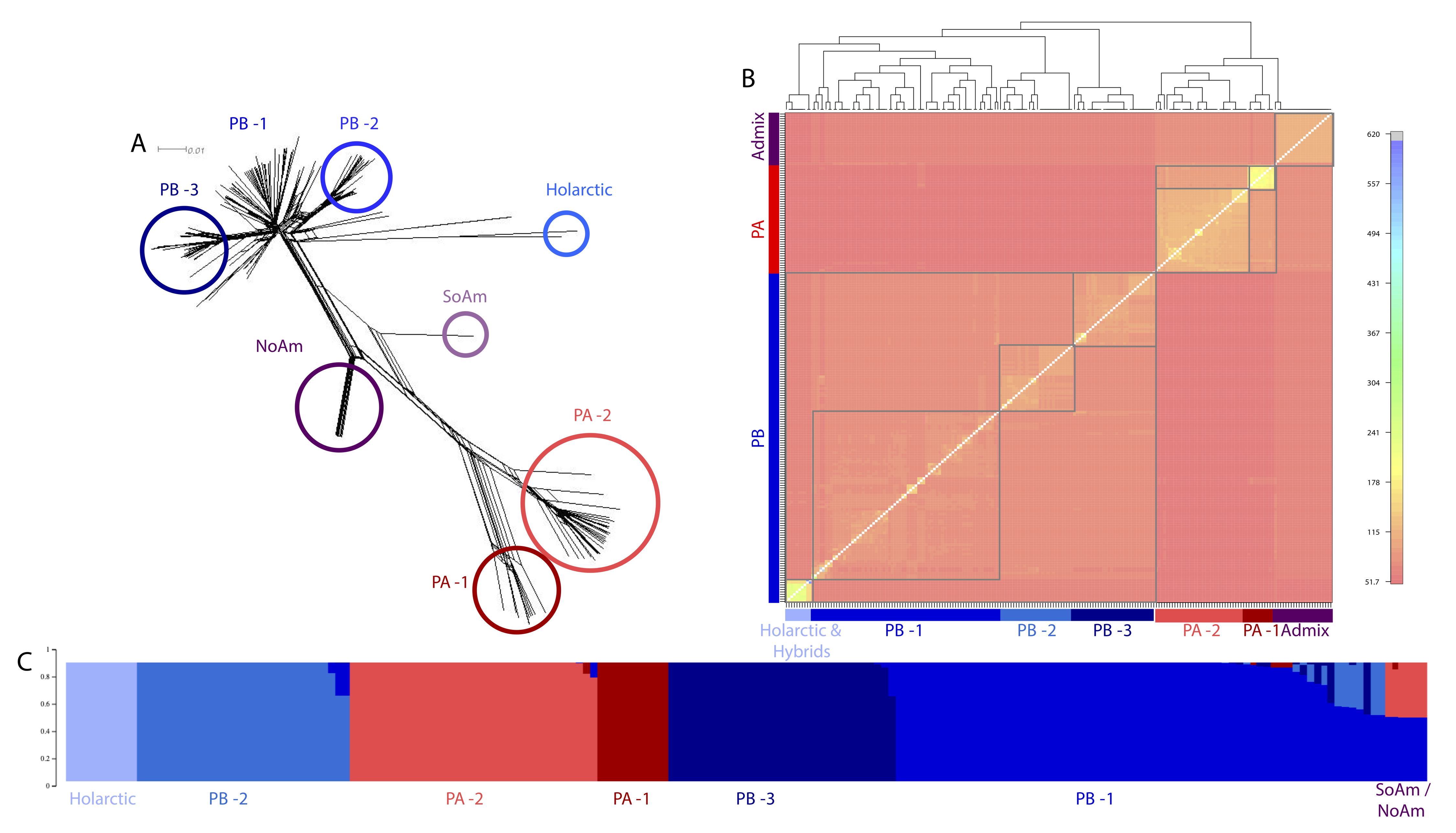

Supplement: S1 Fig — (A) SplitsTree network tree built with 11994 SNPs with subpopulations circled and labeled. (B) FineStructure co-ancestry plot built with 11994 SNPs. Bluer colors correspond to more genetic similarity. Boxes have been added to label the subpopulations. (C) FastSTRUCTURE plot (K = 6) built with 150165 SNPs and showing the same six monophyletic subpopulations found with other approaches. Only five NoAm strains were included in the fastSTRUCTURE analysis. (TIF) [file pgen.1008680.s001.tif]

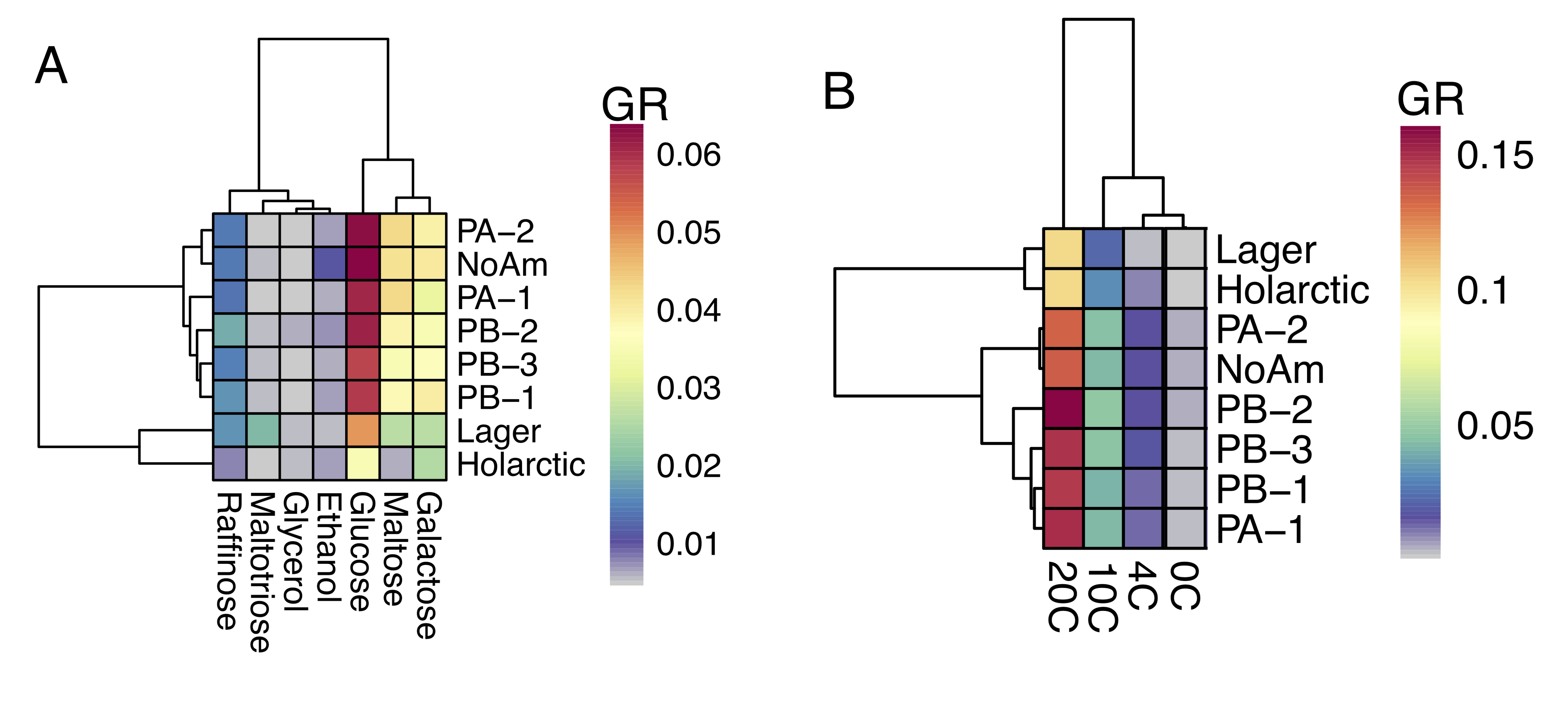

Supplement: S2 Fig — (A) Heat map of mean of maximum growth rate (change in OD/hour) (GR) on different carbon sources by subpopulation. Warmer colors designate faster growth. (B) Heat map of log10 normalized growth at different temperatures by subpopulation. (TIF) [file pgen.1008680.s002.tif]

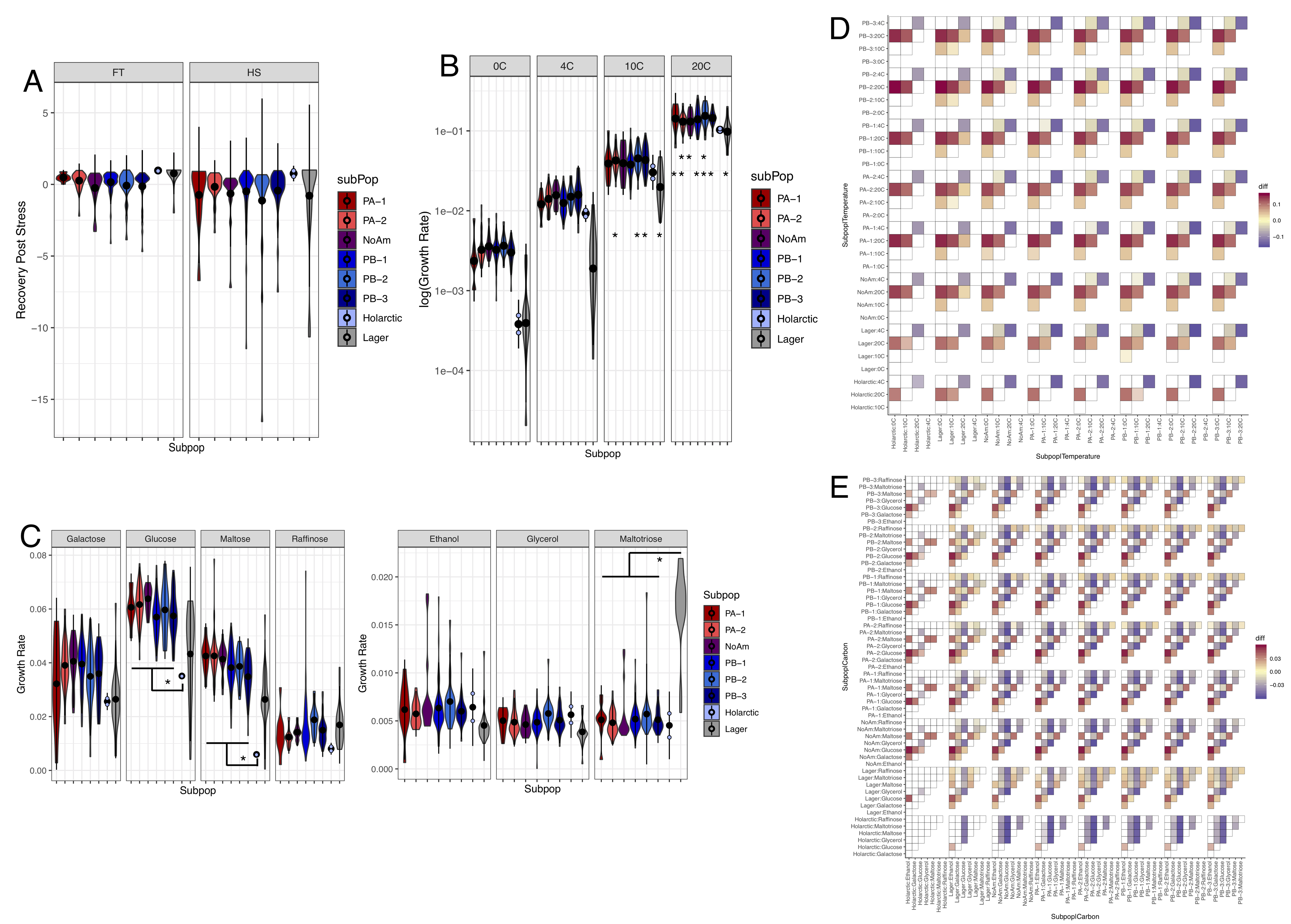

Supplement: S3 Fig — (A) Violin plots of recovery from stress, normalized by controls. There were no significant subpopulation-by-stress interactions. (B) Violin plots of log10 normalized mean growth rates of each subpopulation at 0°C, 4°C, 10°C, and 20°C. * = p-val < 0.05 of interactions between Lager and PA-2, PB-2, and PB-3 at 10°C; Lager and PA-1, PA-2, PB-1, PB-2, and PB-3 at 20°C; and PB-2 and both PA-2 and NoAm at 20°C. (C) Violin plots of mean growth rate on different carbon sources (* = p-val < 0.05). (D) Heatmaps of significant subpopulation-by-temperature interactions and (E) significant subpopulation-by-carbon-source interactions. Warmer colors indicate that the subpopulation-by-temperature or the subpopulation-by-carbon source interactions on the left hand had a faster growth rate than the subpopulation-by-temperature or the subpopulation-by-carbon source along the bottom; cooler colors represent the reverse. Non-significant interactions, based on multiple test corrections, are in white. More intense colors represent smaller p-values. (TIF) [file pgen.1008680.s003.tif]

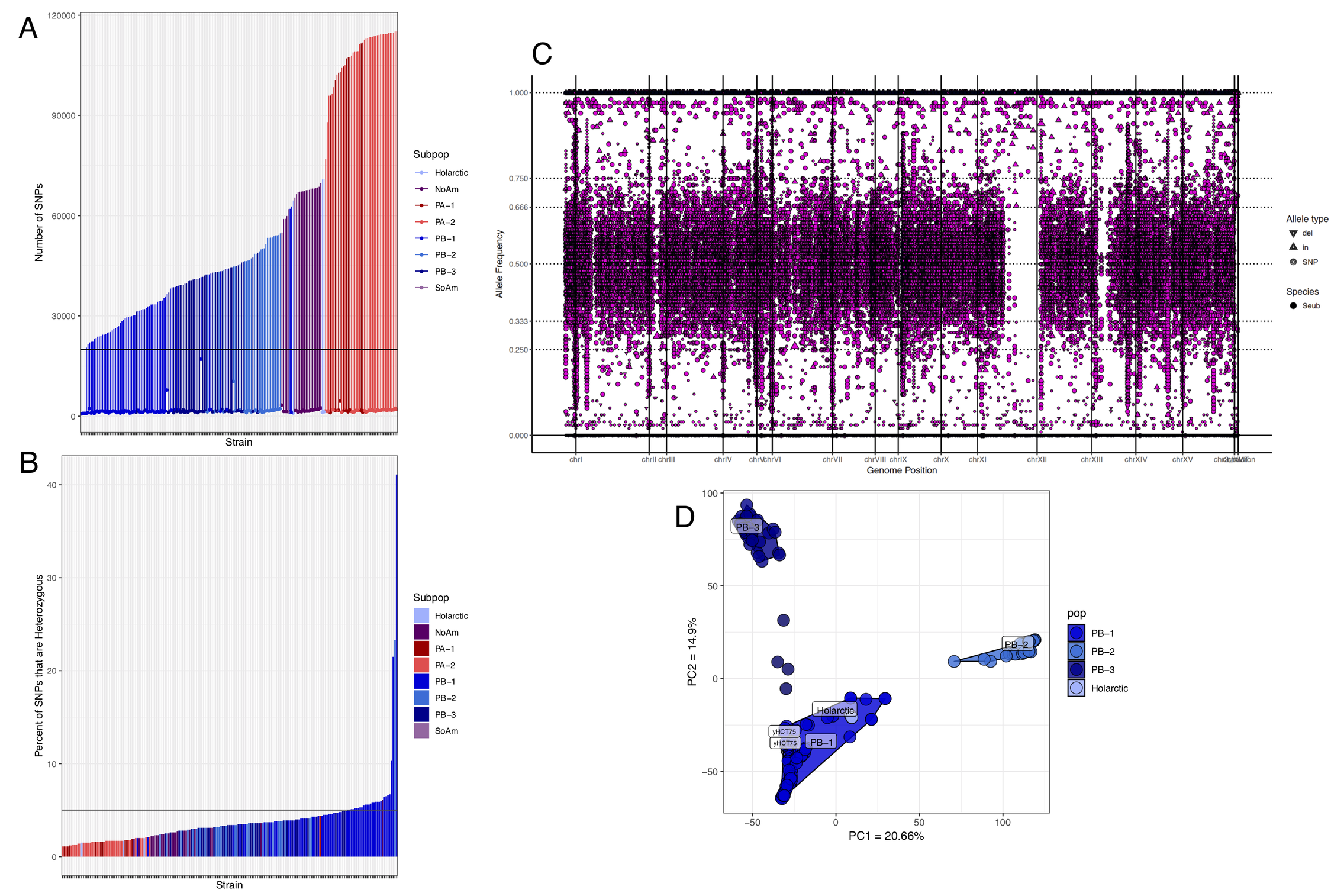

Supplement: S4 Fig — (A) Summary of all SNPs versus SNPs called as heterozygous included in analyses compared to the taxonomic type strain for all pure S. eubayanus strains included in this study. Variants were called on a genome that was not repeat-masked, and strains were subsequently masked for high- and low-coverage regions and for repeats. Shown here are SNP counts after masking for coverage and repeat regions. The upper limit of the bar is the total SNP count. The lower point corresponds to SNPs called as heterozygous. The horizontal line is 20k SNPs. The three strains with low SNP calls (on the left) are derived from the type strain. (B) Percent of SNPs called as heterozygous for all wild pure S. eubayanus strains. The horizontal line is 5% of SNPs called as heterozygous. Most strains have low heterozygosity. (C) Strain yHCT75 (CRUB 1946) is the only strain with > 20K heterozygous SNPs (pre-masking). (D) When the heterozygous SNPs of yHCT75 were pseudo-phased (labeled), both phases clustered with PB-1. (TIF) [file pgen.1008680.s004.tif]

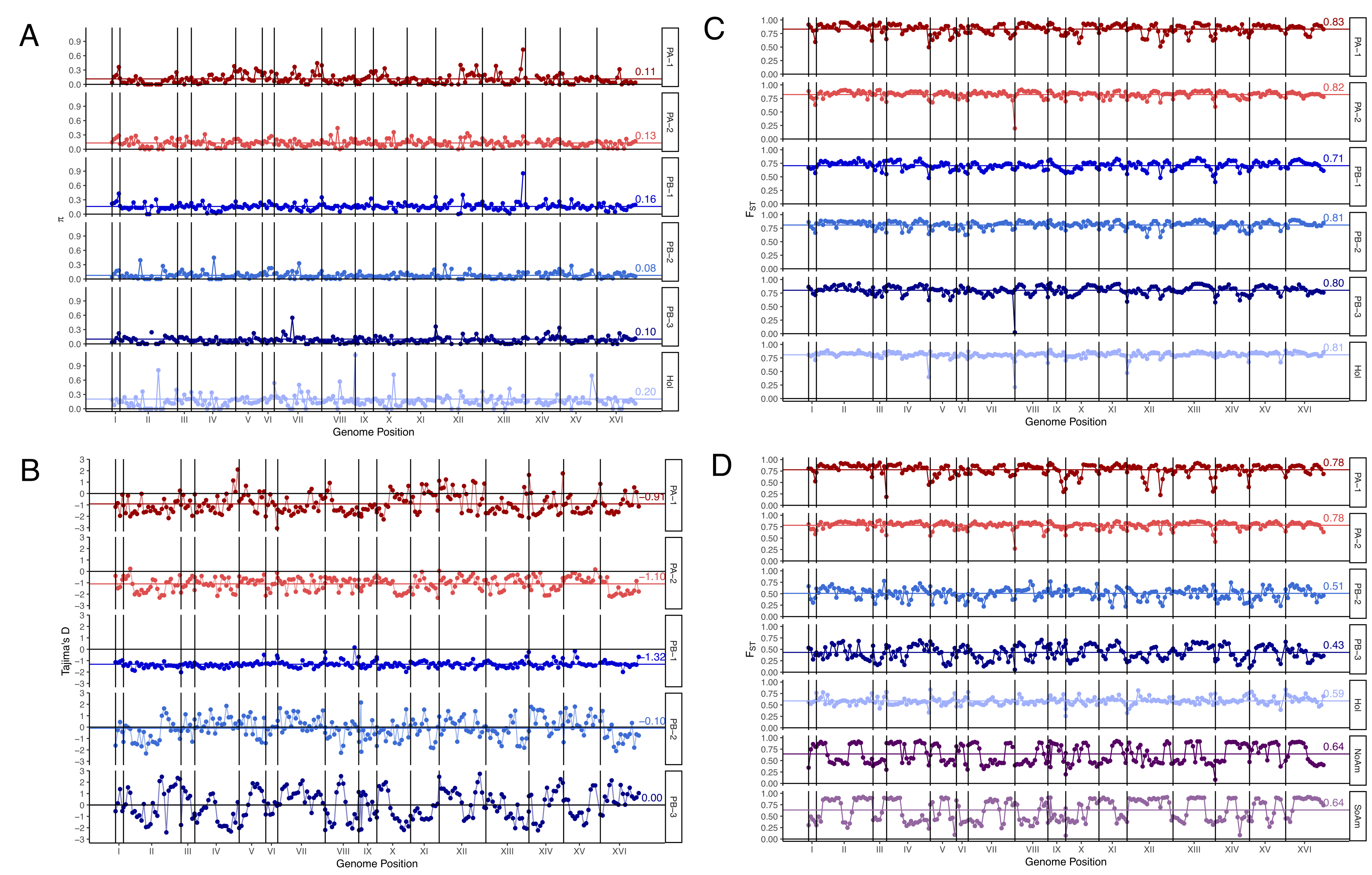

Supplement: S5 Fig — (A) Mean pairwise nucleotide diversity (𝜋 * 100) for each subpopulation across the genome in 50-kbp windows. (B) Tajima’s D across the genome in 50-kbp windows for each subpopulation. (C) Mean FST in 50-kpb windows for each subpopulation compared to all subpopulations. (D) Pairwise FST for each subpopulation compared to PB-1. (TIF) [file pgen.1008680.s005.tif]

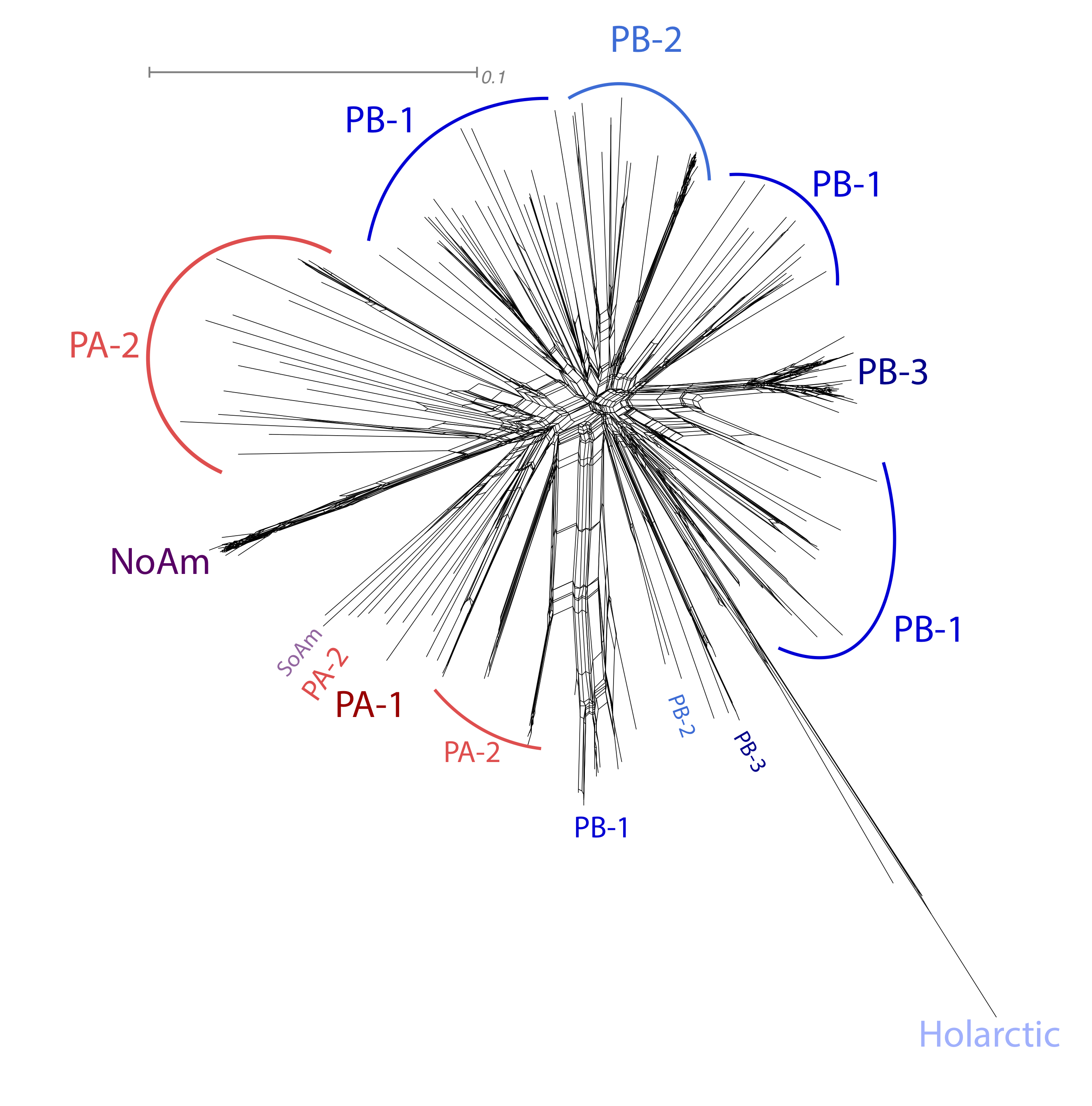

Supplement: S6 Fig — SplitsTree network tree built with 2199 SNPs from the mitochondrial genome. Subpopulations are labeled. Strain yHCT98 was removed due to poor mitochondrial mapping. (TIF) [file pgen.1008680.s006.tif]

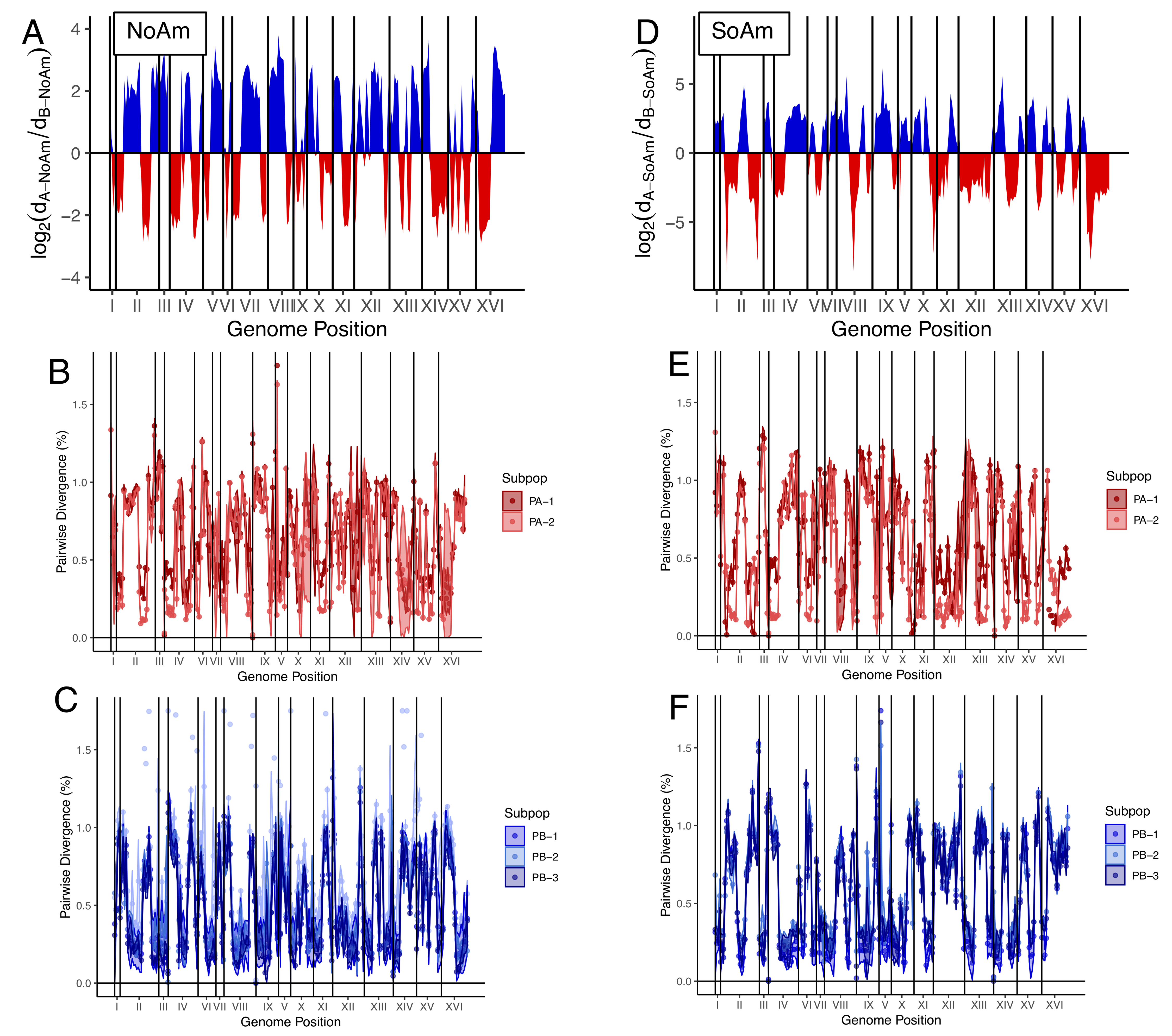

Supplement: S7 Fig — (A) A representative NoAm strain (yHKS210) log2 ratio of the minimum PB-NoAm pairwise nucleotide sequence divergence (dB-NoAm) and the minimum PA-NoAm pairwise nucleotide sequence divergence (dA-NoAm) in 50-kbp windows (adapted from Peris/Langdon et al. 2016) [20]. Colors and log2 < 0 or > 0 indicate that part of the genome is more closely related to PA or PB, respectively. (B) Pairwise nucleotide sequence divergence of the NoAm strain yHKS210 compared to strains from the PA-1 and PA-2 subpopulations of PA in 50-kbp windows. (C) Pairwise nucleotide sequence divergence of the NoAm strain yHKS210 compared to strains from the PB-1, PB-2, and PB-3 subpopulations of PB in 50-kbp windows. (D) log2 ratio of the minimum PB-SoAm pairwise nucleotide sequence divergence (dB-SoAm) and the minimum PA-SoAm pairwise nucleotide sequence divergence (dA-SoAm) in 50-kbp windows. Colors are as in A. (E) Pairwise nucleotide sequence divergence of the SoAm strain compared to strains from the PA-1 and PA-2 subpopulations of PA in 50-kbp windows. (F) Pairwise nucleotide sequence divergence of the SoAm strain compared to strains of the PB-1, PB-2, and PB-3 subpopulations of PB in 50-kbp windows. (TIF) [file pgen.1008680.s007.tif]

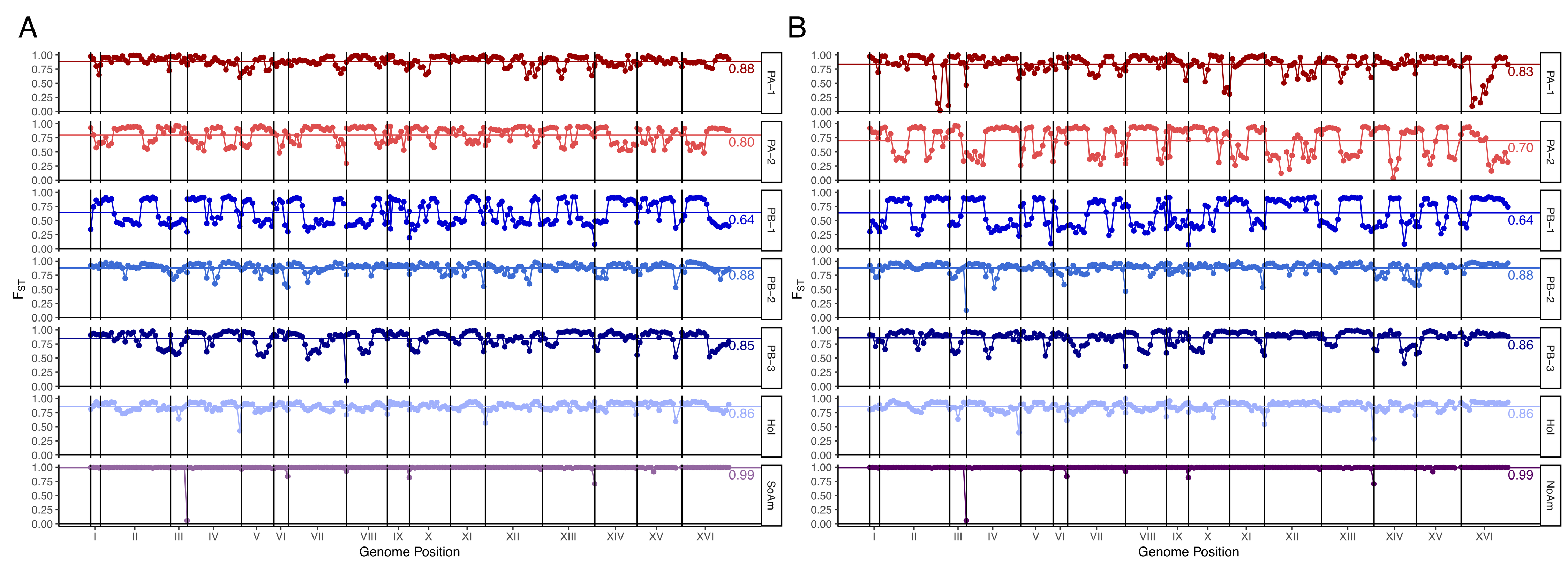

Supplement: S8 Fig — Pairwise FST for the NoAm lineage (A) or SoAm strain (B) compared to all other subpopulations. (TIF) [file pgen.1008680.s008.tif]

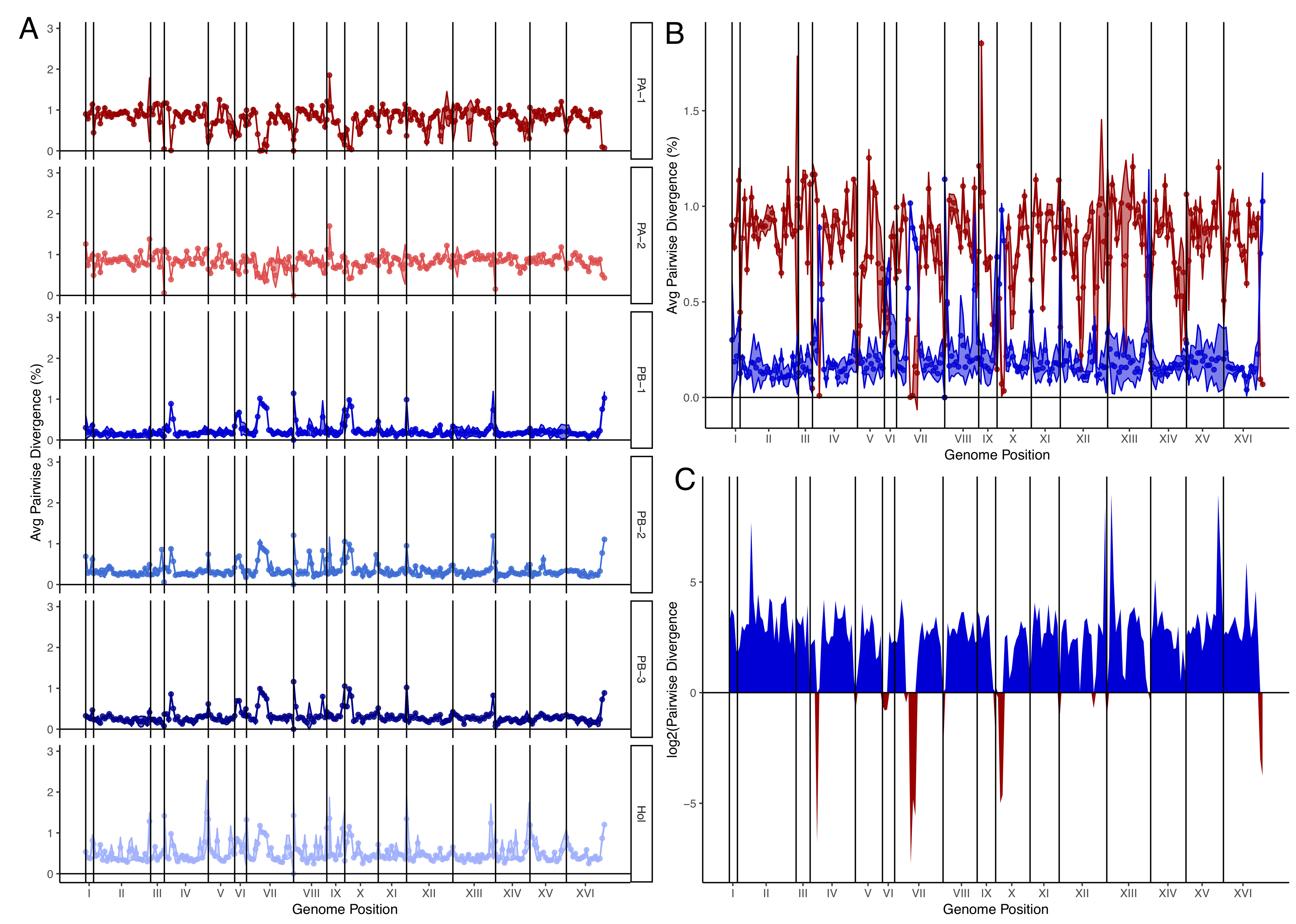

Supplement: S9 Fig — (A) Pairwise genetic divergence of the taxonomic type strain compared to each subpopulation. (B) Comparison of pairwise genetic divergence of the taxonomic type strain compared to PA-1 and PB-1. (C) log2 divergence plot (as in Fig 4) showing regions introgressed from PA-1 in the taxonomic type strain. (TIF) [file pgen.1008680.s009.tif]

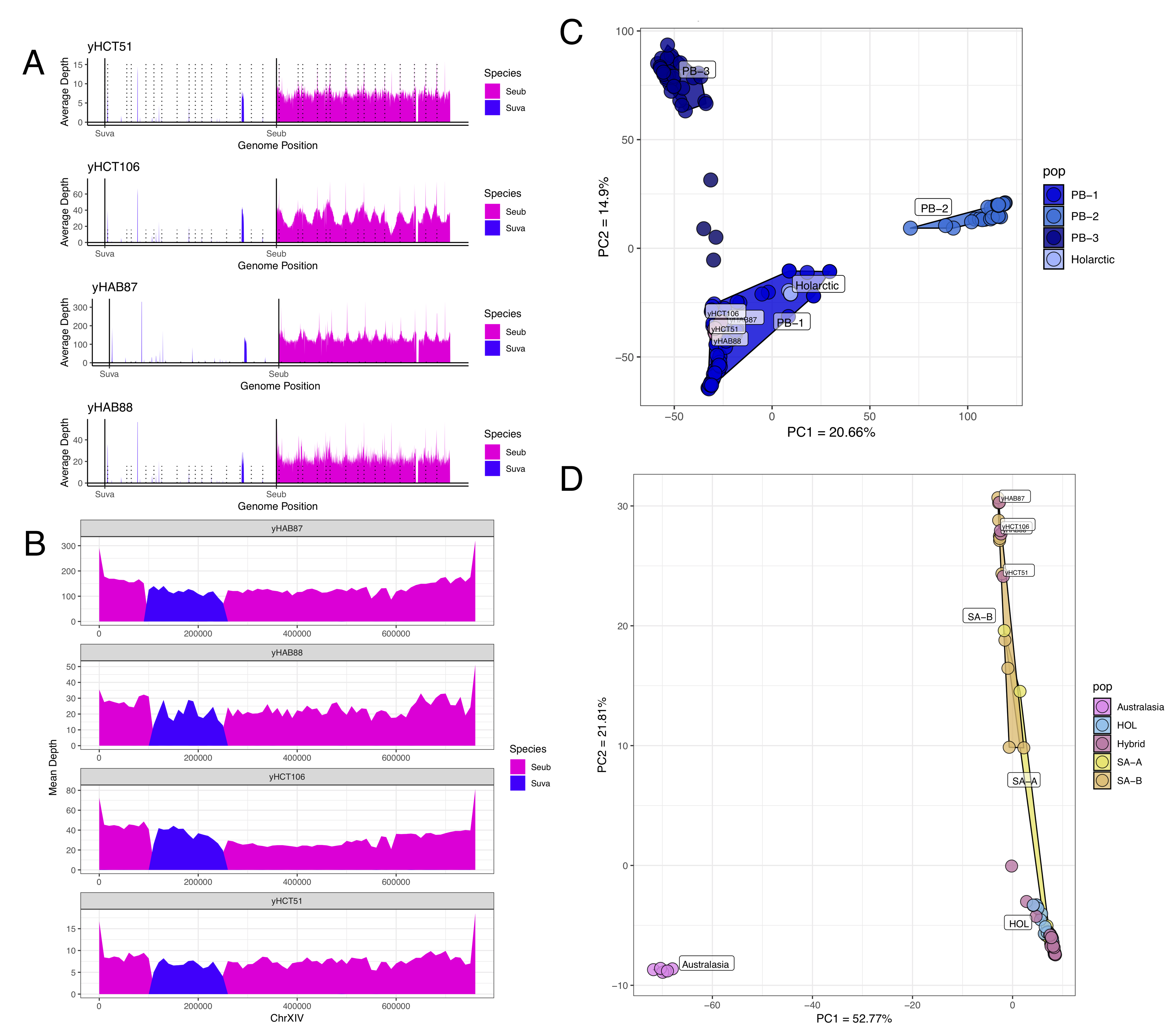

Supplement: S10 Fig — (A) Depth of coverage plots of reads from four strains mapped to both the S. uvarum (Suva) and S. eubayanus (Seub) reference genomes. (B) Zoom-in of region on Chromosome XIV where these four strains have the same S. uvarum (purple) introgression into a S. eubayanus background. (C) A PCA plot shows that these four strains belong to the PB-1 subpopulation of S. eubayanus. (D) A PCA plot shows that the introgressed region from S. uvarum came from the South American SA-B subpopulation of S. uvarum. (TIF) [file pgen.1008680.s010.tif]

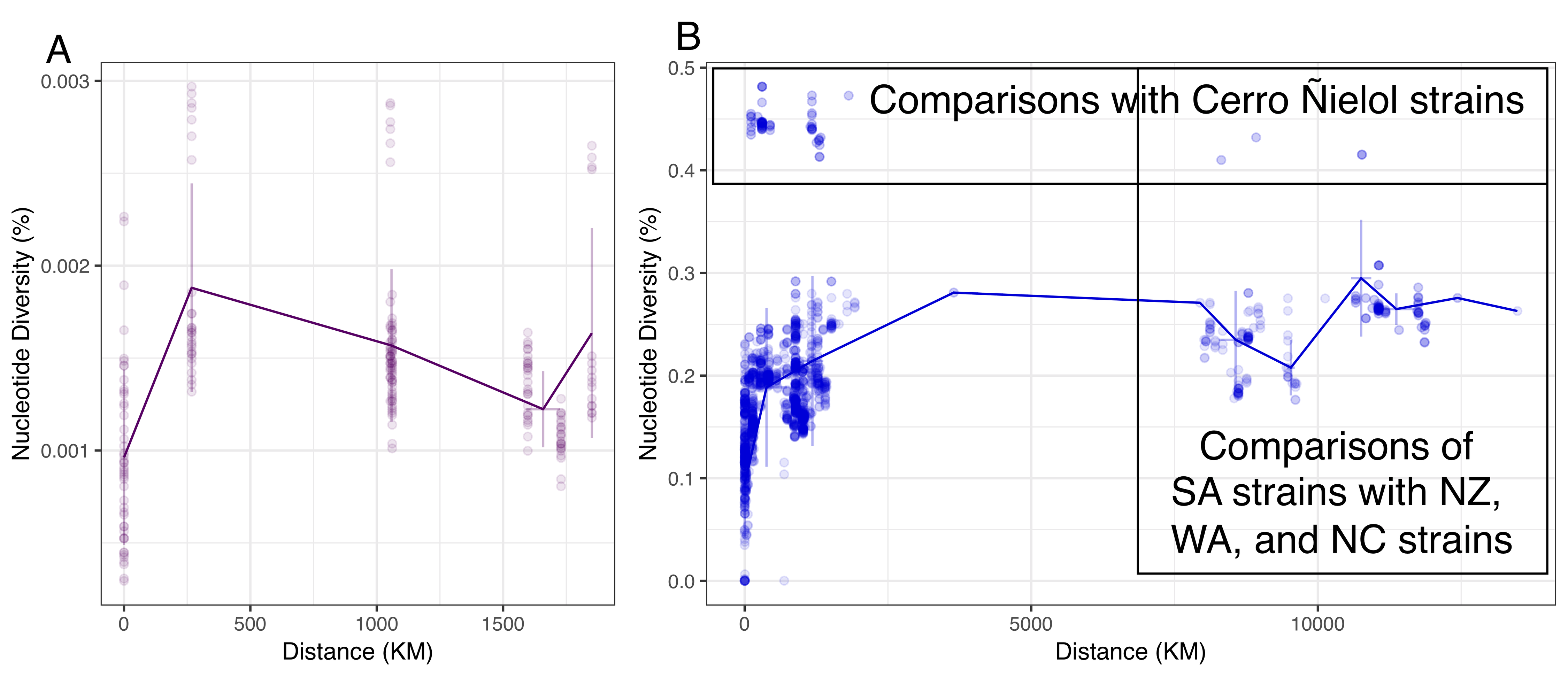

Supplement: S11 Fig — (A) Isolation by distance for all NoAm strains. The y-axis has been rescaled compared to Fig 5 for better visualization. (B) Isolation by distance for subpopulation PB-1. Comparisons with strains from Cerro Ñielol are labeled. All comparisons of South American strains with non-South American strains are on the right side. (TIF) [file pgen.1008680.s011.tif]

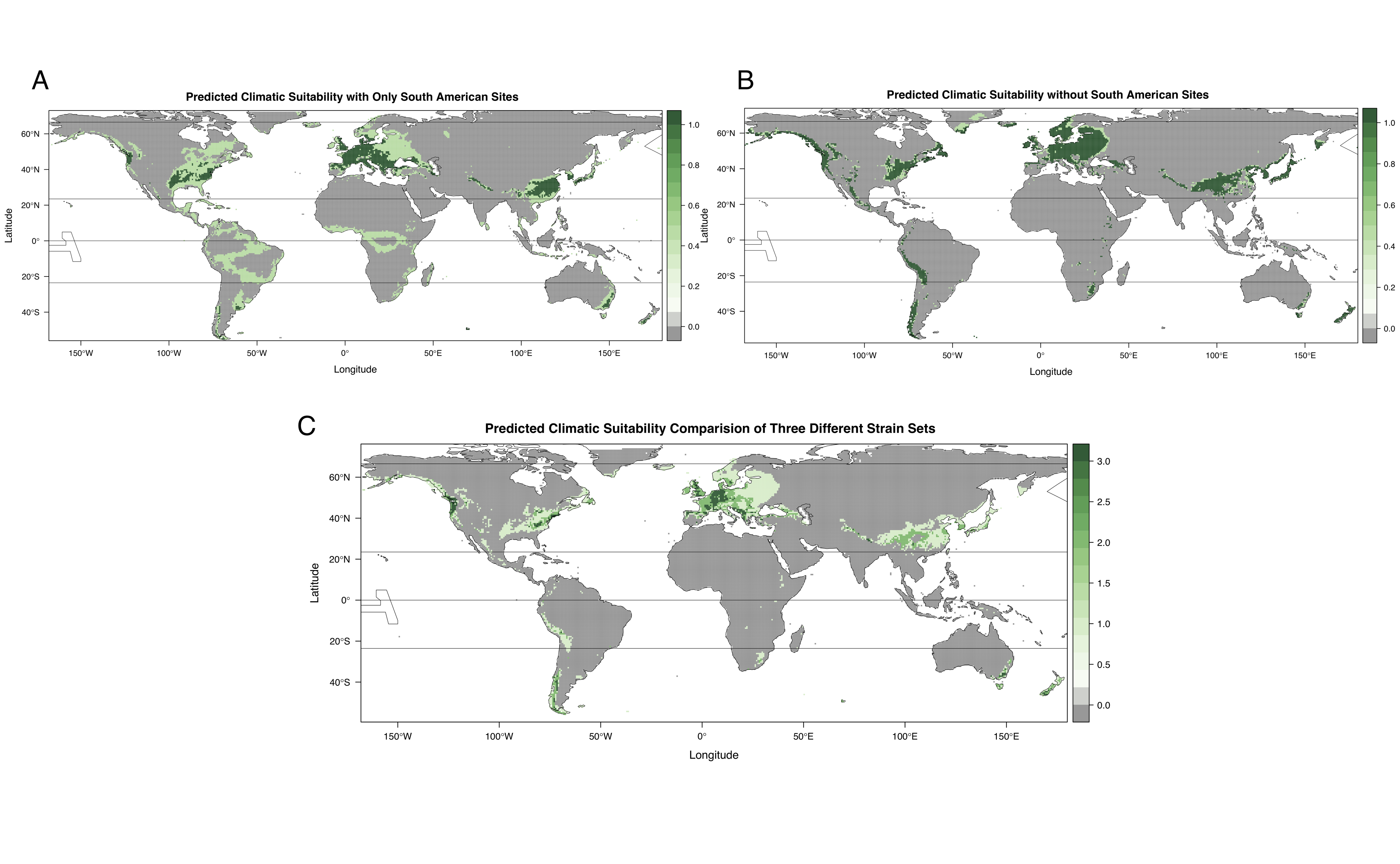

Supplement: S12 Fig — (A) Model built using only South American isolation locations. (B) Model built using only non-South American sites. (C) Comparison of models based on all known S. eubayanus collection sites, only South American, or only non-South American sites. Where the models agree is in dark green, where two models agree is in medium green, and where one model predicts suitability is in light green. (TIF) [file pgen.1008680.s012.tif]
